# Short-term effects of cannabis legalisation in Germany on driving under the influence of cannabis: a difference-in-differences analysis using Austria as a control

**DOI:** 10.1016/j.lanepe.2026.101593

**Published:** 2026-01-23

**Authors:** Anna Schranz, Anja Knoche-Becker, Moritz Rosenkranz, Uwe Verthein, Jakob Manthey

**Affiliations:** a Centre for Interdisciplinary Addiction Research of Hamburg University (ZIS), Department of Psychiatry and Psychotherapy, University Medical Centre Hamburg-Eppendorf (UKE), Martinistraße 52, 20246, Hamburg, Germany; b Federal Highway and Transport Research Institute (BASt), Brüderstraße 53, 51427, Bergisch Gladbach, Germany; c Department of Psychiatry, Medical Faculty, University of Leipzig, Semmelweisstraße 10, 04103, Leipzig, Germany

**Keywords:** Driving under the influence, Cannabis, Legalisation, Germany, Cross-sectional studies, Road safety impact

## Abstract

**Background:**

In April 2024, Germany legalised adult cannabis possession and cultivation, and in August 2024 established legal THC-limits for driving. This study aimed to examine short-term impacts on (1) cannabis use and (2) driving under the influence of cannabis (DUIC), and (3) investigates the extent of DUIC involving cannabis combined with alcohol or other drugs (DUIC(+)).

**Methods:**

Data came from two cross-sectional population surveys in Germany and Austria (control) before (t_0_: Nov–Dec 2023) and after legalisation (t_1_: Nov 2024–Jan 2025). We assessed 12-month cannabis use among adults aged 18–64 (Germany: n_t0_ = 6670, n_t1_ = 9692; Austria: n_t0_ = 2132, n_t1_ = 2102) and DUIC among at least monthly cannabis users (excluding medical use; Germany: n_t0_ = 393, n_t1_ = 589; Austria: n_t0_ = 86, n_t1_ = 92) using a difference-in-differences (DiD) approach. For t_1_, we compared the proportion of DUIC(+) and cannabis-only DUIC(−) episodes among all DUIC episodes by use frequency.

**Findings:**

In Germany, cannabis use rose from 12·1% to 14·4%, but this trend did not significantly differ from Austria (DiD-effect: *OR* = 1·18, 95% CI 0·95–1·48, *p* = 0·141, weighted). Among at least monthly users, DUIC decreased slightly from 28·5% to 26·8% (unweighted), with no significant difference compared with Austria (DiD-effect: *aOR* = 0·68, 95% *CI* 0·27–1·68, *p* = 0·408). Results held across sensitivity analyses including additional confounders and negative controls. At t_1_, DUIC(+) accounted for 21·5% of episodes. DUIC(−) was most common among daily users, DUIC(+) among weekly users.

**Interpretation:**

Eight months after legalisation, no significant short-term effects on cannabis use or DUIC were observed. DUIC(+), associated with higher traffic risk, was most common among weekly users. A comprehensive evaluation of the cannabis reform requires further monitoring of DUIC and traffic data.

**Funding:**

Federal Highway and Transport Research Institute (FE 82.0816/2023).

**Research in context**

**Evidence before this study**

Four databases (Web of Science, Psyndex, PubMed, and Scopus) were searched for studies that examined DUIC or crash-related outcomes following recreational cannabis legalisation using a combination of search terms (“(traffic OR accident OR crash OR DUIC OR driving under the influence of cannabis) AND (cannabis OR marijuana OR THC OR tetrahydrocannabinol) AND (legalisation OR legalisation OR legal) NOT (animal OR rat OR mice)”). The search was limited to original research published between 1st of January 2010 and 31st of December of 2023 in English or German language. An update of the search was conducted on 15th of October 2025.

We identified publications mainly from North America; apart from one regionally limited study from southwest Saxony (Germany), no evidence from Europe is available. Some but not all studies point to increased DUIC following cannabis legalisation. While DUIC prevalence appears to be generally higher among more frequent users, previous studies have not controlled for co-use of other substances in addition to cannabis before driving (e.g., alcohol), making it impossible to delineate the traffic safety impact of cannabis from other substance use.

For the impact on motor vehicle crashes, the evidence is more mixed: some report no effect, while others suggest an increase in the number of all-cause fatalities of low to medium magnitude.

**Added value of this study**

This study considers two mechanisms that can contribute to changes in DUIC after cannabis legalisation through two indicators: 1) changes in prevalence of use in the general population and 2) changes in DUIC among those who use cannabis. The results suggest that both indicators remained stable after cannabis legalisation in Germany after controlling for secular trends with data from Austria, where cannabis remained illegal.

Moreover, four in five DUIC episodes were reported to involve only cannabis, while the remaining DUIC episodes also involved other substances. Considering the distribution of DUIC episodes by concurrent substance use and by cannabis use frequency, explorative analyses tentatively suggest that the largest risk for traffic safety may arise from people using cannabis weekly, but not daily.

**Implications of all the available evidence**

This study is the first nationwide evaluation of cannabis legalisation in a European country. The results show neither a significant short-term shift in cannabis use prevalence nor in the prevalence of DUIC among cannabis users following legalisation in Germany. While further monitoring of possible negative consequences of cannabis legalisation is required, the German cannabis legalisation model appears to broadly align with public health goals with respect to prevalence of use and traffic safety.

## Introduction

Internationally, recreational cannabis legalisation has expanded in recent years (e.g., several U.S. states since 2012, Uruguay in 2013, Canada in 2018). In April 2024, Germany followed suit by legalising the possession and cultivation of cannabis for recreational use, but it upheld the ban on commercial cannabis sales. At the same time, medical cannabis access was liberalised by removing it from the narcotics law.^1^ While the liberalisation of cannabis policies is often aimed at improved regulation, reduced harms, and a kerbed illicit market, it has also raised public health concerns, including a potential increase in driving under the influence of cannabis (DUIC) and related crash risk (alongside possible increases in use among youth, mental health problems, and cannabis-related emergency department visits). Cannabis use can impair driving ability^2^ and a recent meta-analysis suggests DUIC is associated with increased risk of motor vehicle crashes (MVCs), including higher odds of fatality (OR 1·55) and injury (OR 2·00), corresponding to 14 additional deaths per 100,000 MVCs and a 6·8% increase in injuries.^3^

The evidence on the impact of cannabis legalisation on DUIC remains inconclusive.^4^ While some studies report increases in DUIC following legalisation (e.g., Adhikari et al.^5^) or in states with less restrictive cannabis policies,^6^ others find no clear association (e.g., Walker et al.^7^) or even decreases (e.g., Voy^8^), likely highlighting the complexity of regulatory, cultural, and enforcement factors influencing driving behaviour.

In Germany, cannabis use is widespread and increasing. A 2021 national survey found that 8·8% of adults aged 18–64 reported cannabis use in the previous year, representing nearly a doubling since 2012.^9^ According to the same survey, about 3% of people using cannabis in the past year also reported DUIC.^10^ In August 2024, Germany increased the legal limit of tetrahydrocannabinol (THC) in road traffic from 1 to 3·5 ng (ng) per millilitre of blood serum. For novice drivers, those under the age of 21 years and the mixed use with alcohol, the previous limit of 1 ng THC/ml blood serum remains in place (German Road Traffic Act, StVG § 24a and 24c). In Austria, no THC limits apply; legal consequences depend on impairment (Austrian Road Traffic Act, StVO § 5 (1)).

This study seeks to evaluate possible short-term changes in DUIC following cannabis legalisation in Germany. Changes in DUIC may be the result of:

(a) Changes in the number of people who use cannabis; and/or
(b) Changes in the proportion of users who engage in DUIC.

These mechanisms carry distinct implications for policy: while (a) reflects broader use trends, (b) implies individual behavioural adaptation to legal change, e.g., in response to the new THC-limit. Such adaptation may be shaped by processes of normalisation^11^—where DUIC becomes more socially accepted—or deterrence,^12^ whereby intensified enforcement or perceived detection risk discourages DUIC.

Many previous studies did not address mechanisms (a) and (b). For example, higher rates of positive tests for THC in drivers post-legalisation^13^ may simply reflect increased use prevalence (mechanism (a)) rather than changes in individual traffic safety behaviour (mechanism (b)). Recent evidence from Canada supports mechanism (a): increases in DUIC prevalence among the general population following legalisation can be attributed to greater overall cannabis use, whereas DUIC prevalence among cannabis users remained stable or even declined.^14^ This underscores the importance of differentiating between the two mechanisms. Accordingly, we analysed trends in (a) cannabis use in the general population as well as (b) DUIC among at least monthly users. The following pre-registered hypotheses guided our research:

**H1**: The legalisation of cannabis in Germany is followed by a greater increase in cannabis use prevalence compared with Austria (control region).

**H2**: The legalisation of cannabis in Germany is *not* followed by an increase in (self-reported) DUIC prevalence among at least monthly cannabis users.

In the literature (see Methods, subsection ‘secondary outcome: DUIC’ for common definitions), self-reported DUIC is usually defined as driving a motor vehicle within a few hours after using cannabis. This definition does not acknowledge two aspects that are presumably crucial to understanding the crash risk linked to DUIC: First, if cannabis is used in combination with other substances, the impairment increases substantially (for alcohol, see e.g., Simmons et al.^15^). Second, more frequent users may experience less impairment from the same dose of THC due to tolerance,^16^ but they may engage in greater DUIC frequency^17^–with unknown implications for traffic safety. In addition to testing the legalisation-related hypotheses, we further aimed to accommodate these two aspects to better understand the traffic safety implications of DUIC in Germany and Austria.

## Methods

### Study population and data collection

In a quasi-experimental difference-in-differences (DiD) study design, we conducted a repeated cross-sectional survey with two measurement points in Germany (intervention) and Austria (control: no change in cannabis policy), before and after cannabis legalisation in Germany. Austria was considered a suitable control, as past 12-month cannabis use (see Figure S1.1) and drug-impaired MVCs (see Figure S1.2) have increased comparably in both Austria^18^ and Germany^9^ over recent years. For a discussion of the parallel trends assumption underlying the DiD and its empirical assessment, see Supplement S1.

Data were collected at baseline (t_0_: Nov–Dec 2023) and follow-up (t_1_: Nov 2024–Jan 2025) via computer-assisted web interviewing (CAWI). Data from a second follow-up, planned for Dec 2025, will be included in future analyses. Participants between 18 and 64 years were recruited via an ISO-certified online access panel using both online and offline recruitment (Table S2) and received financial reimbursement by the panel provider (www.bilendi.de). Samples were quota-sampled on age, gender, education, and federal state, reflecting the demographic composition of residents aged 18–64 of each country. To reduce self-selection bias related to cannabis use (i.e., overrepresentation of participants with strong opinions on cannabis), the study focus on cannabis was not mentioned in the survey invitation. Because representativeness in modern substance use surveys is difficult to achieve,^19^ we assessed the sample composition in depth (for geographic and socioeconomic distribution see Figure S3 and Table S3.1, respectively) and validated against probability-based national surveys (Table S3.2).

Overall, *n* = 127,552 individuals were invited, of whom *n* = 30,650 respondents (24·3%) accessed the survey link. A total of *n* = 13,593 (44·3%) respondents who accessed the link completed the survey. Participants with unrealistic short response times, low response variability, or failing plausibility and control checks were excluded (for details on response metrics and sample preprocessing, see Figure S2). To ensure sample independence, individuals participating in both t_0_ and t_1_ were also excluded from both samples.

At t_0_, *n* = 6670 respondents in Germany and *n* = 2132 in Austria completed the initial survey questions on cannabis use; at t_1_, *n* = 9692 and *n* = 2102 respectively. Respondents reporting any cannabis use in the past 12 months (t_1_) or at least monthly use (t_0_; reflecting an update in filtering criteria between survey waves) were directed to the extended cannabis module, which included questions on DUIC.

**Fig. 1:**
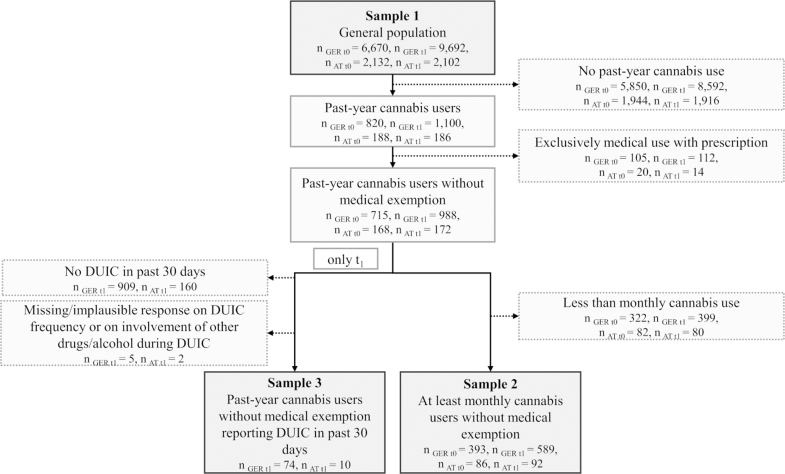
**Flow chart of sample selection for Sample 1 (general population), Sample 2 (at least monthly cannabis users without medical exemption), and Sample 3 (past-year cannabis users without medical exemption reporting DUIC in the past 30 days).** Unweighted absolute sample sizes are reported. DUIC, driving under the influence of cannabis; t_0_: Nov–Dec 2023, t_1_: Nov 2024–Jan 2025; GER, Germany, AT, Austria.

We defined three analytical samples (for an overview, see Fig. 1):

• **Sample 1** (general population, t_0_ and t_1_): all respondents, irrespective of cannabis use. This sample was used for estimating the effect of legalisation on cannabis use prevalence (H1).
• **Sample 2** (at least monthly cannabis users without medical exemption, t_0_ and t_1_): respondents with medical-only use with a prescription were excluded (see Fig. 1 for the number of cases excluded), as they are legally permitted to drive after using medical cannabis in Germany if using the medication as prescribed (German Road Traffic Act, StVG §24a (4)). Since only respondents with monthly or more frequent cannabis use were asked DUIC-related questions at t_0_, we applied the same inclusion criterion at t_1_ to ensure comparability. This sample was used for estimating the effect of legalisation on DUIC prevalence (H2).
• **Sample 3** (past-year cannabis users without medical exemption reporting DUIC in the past 30 days, t_1_ only): respondents reporting cannabis use in the last 12 months and DUIC within the past 30 days at t_1_, used to explore the distribution of DUIC episodes by cannabis use frequency and by the involvement of cannabis in combination with alcohol or other drugs (DUIC(+)) versus cannabis alone (DUIC(−)). Only cases with complete information on the number of DUIC episodes and on the co-use of alcohol or other drugs were included. t_0_ data were excluded due to insufficient granularity on the number of DUIC episodes and DUIC(+)/(−). The focus on DUIC within the past 30 days (rather than 12 months) was chosen to reduce potential recall bias associated with longer reference periods. Data from Germany and Austria were combined for analysis (for a sensitivity analysis using German data only, see Figure S11.1).

Further methodological details are provided in the published study protocol.^20^

### Measures

The full questionnaires are publicly available (https://osf.io/72nyf/), and the wording of items used in the analyses is provided in the Table S4.

#### Primary outcome: cannabis use

Cannabis use in the past 12 months was a binary outcome variable (0 = never; 1 = less than monthly/monthly/weekly/ (near) daily; Item ‘SUBSTANCES’).

#### Secondary outcome: DUIC

DUIC was defined as having driven a vehicle within 2 h after using cannabis in the past 12 months (0 = ‘No, never’ or ‘Yes, more than 12 months ago’; 1 = ‘Yes, within the past 30 days’ or ‘Yes, within the past 12 months’; Item ‘DUIC’). Many previous studies have defined DUIC as driving within 1 to 4 h after cannabis use (e.g., Germany: Epidemiological Survey of Substance Abuse (≤2 h) and European Web Survey on Drugs (≤4 h); Canada: National Cannabis Survey (≤2 h), Canadian Cannabis Survey (≤2 h post-use (2018), or ≤2 h post-inhalation/≤4 h post-ingestion (2019–2023)) and CAMH Monitor (≤1 h); International Cannabis Policy Study ≤2 h). By adopting the 2-h timeframe, we aligned with commonly used definitions in the literature and focused on the period of highest impairment risk and THC peak levels, particularly relevant for the most common use methods, such as smoking or vaporising.^21^^,^^22^

To address potential (time-varying) measurement biases in self-reported DUIC due to (a) social desirability (e.g., changes in stigma post-legalisation), we examined the association of DUIC with the trait ‘Minimisation of Negative Qualities’ (NQ–; a subscale of the Social Desirability—Gamma Short Scale, KSE-G^23^; see Table S4 for item wording), assessed whether this association changed after legalisation, and additionally tested for changes in self- and perceived stigmatisation toward people using cannabis (see Supplement S5). Further, we addressed (b) recall bias by additionally analysing past 30-day DUIC (shorter recall period).

#### Additional outcomes: DUIC(−) and DUIC(+) episodes

As polysubstance use—particularly the combination of cannabis and alcohol—is frequently observed among THC-positive drivers involved in traffic crashes^24^^,^^25^ and using cannabis in combination with alcohol or other drugs before driving poses greater safety risks,^15^ we conducted exploratory analyses to estimate how many DUIC episodes involved (a) cannabis only (DUIC(−)) versus (b) cannabis in combination with alcohol or other drugs (DUIC(+)). This differentiation was based on two items presented to participants who reported DUIC, asking (1) how frequently they co-used alcohol or other drugs when driving after cannabis use (‘Cannabis only’, ‘Occasionally used alcohol or other drugs in addition to cannabis’, ‘Mostly used alcohol or other drugs in addition to cannabis’, ‘Always used alcohol or other drugs in addition to cannabis’; Item ‘DUIC_MIXEDUSE’; assessed at t_1_ only) and (2) the number of DUIC episodes in the past 30 days (‘once’, ‘2–3 times’, ‘4–9 times’, ‘10–15 times’, exact frequency entered if ‘more than 15 times’; categories were converted into numeric values by using the midpoints of the intervals, e.g., ‘2–3 times’ was coded as 2·5). Since the co-use of alcohol or other drugs was assessed categorically rather than per individual DUIC episode, we translated the verbal response options into numeric proportions to approximate the number of DUIC(−) and DUIC(+) episodes per person. Specifically, we assumed the following distributions: ‘Cannabis only’ = 100% of the DUIC episodes classified as DUIC(−), ‘Occasionally used alcohol or other drugs in addition to cannabis’ = 75% DUIC(−), ‘Mostly used alcohol or other drugs in addition to cannabis’ = 25% DUIC(−), and ‘Always used alcohol or other drugs in addition to cannabis’ = 0% DUIC(−). The remaining proportion of DUIC episodes was assigned to DUIC(+). Sensitivity analyses using different quantifications of ‘Occasionally’ and ‘Mostly’ are provided in the Supplement S11.

#### Covariates

Sociodemographic covariates included age (18–24, 25–34, 35–44, 45–64 years), gender (male, female), driver's licence status (1 = yes, 0 = no), degree of urbanisation (city, towns/suburbs, rural, according to DEGURBA,^26^ using municipality codes—directly for Germany, and via ZIP-to-municipality mapping for Austria), and education. Educational levels were classified according to ISCED 11^27^ into low (ISCED levels 0–2), medium (ISCED levels 3–4), and high (ISCED levels 5–8) categories. There were *n* = 77 non-binary participants (0·03% of pooled sample), who were randomly assigned to the male or female category in order to avoid empty or sparse cells.

Behavioural covariates included frequency of gambling, cannabis (only relevant for DUIC model), and alcohol use (as indicators for risk-taking behaviour and substance use proneness) and of physical activity (as a proxy for health behaviour). All variables were coded categorically with the levels ' (near) daily’, ‘at least weekly’, ‘at least monthly’, ‘less than monthly’, ‘never’ (item ‘SUBSTANCES'; for cannabis use frequency, only the categories ‘at least monthly’ and higher were assessed).

These variables were screened for bivariate associations with cannabis use or DUIC in the baseline sample (t_0_, pooled across countries) using chi-square tests. Variables with *p* < 0·10 were included in the adjusted models (Supplement S6).

### Analyses

The DiD analyses followed the pre-registered study protocol^20^; sensitivity analyses and the separation of DUIC(−) and DUIC(+) were not pre-planned. All analyses were performed using *R* version 4·4·0.

#### Difference-in-differences analyses

To assess the impact of legalisation in Germany, we employed a DiD-design comparing changes in (1) cannabis use and (2) DUIC prevalence in Germany to changes observed in Austria, where no policy change occurred (cannabis possession and cultivation is a criminal offence in Austria). By using Austria as a control group, we were able to account for temporal confounding factors that may have influenced both countries around the time of the policy change. The central assumption of the DiD-framework is that, in the absence of the legalisation, trends in the outcome variables in Germany would have followed a parallel trajectory to those in Austria. We conducted post-hoc simulations to estimate the minimum detectable DiD-effect at 80% power (α = 0·05) for cannabis use and DUIC (see Supplement S7).

**Cannabis use (H1):** DiD analysis was conducted using a logistic regression model with the outcome variable cannabis use in the past 12 months (0 = no, 1 = yes). Predictors included time (0 = 5–6 months before legalisation (t_0_), 1 = 7–9 months after legalisation (t_1_)), country (0 = Austria, 1 = Germany), and their interaction (time × country). A statistically significant interaction term (time × country) would suggest a differential change in the outcome in Germany relative to Austria, potentially attributable to the legalisation. Statistical significance was set at α = 0·05 (two-tailed), as changes in either direction are theoretically meaningful. Post-stratification weights were applied to align the distribution of Sample 1 with the general population of Germany in terms of age, gender, education, and federal state (for details of the weighting procedures, see Supplement S8).

As robustness checks, we conducted sensitivity analyses using alternative model specifications and a negative control. Specifically, in addition to the main model, we estimated (1) an unweighted model without covariates, (2) unweighted model including sociodemographic covariates, (3) an unweighted model including both sociodemographic and behavioural covariates, (4) a negative control model using past 12-month fish consumption as the outcome, which should be unaffected by cannabis legalisation (for rationale and negative control selection, see Supplement S9), and (5) a weighted model using German data only to assess changes in cannabis use independent of the cross-country comparison.

We reported unadjusted odds ratios (ORs) from the primary (weighted) DiD model; for adjusted odds ratios (aORs), see Table S9.1.

**DUIC (H2):** DiD analysis was conducted using an unweighted logistic regression model with the outcome variable DUIC in the past 12 months (0 = no, 1 = yes). Predictors included time (0 = 5–6 months before legalisation (t_0_), 1 = 7–9 months after legalisation (t_1_)), country (0 = Austria, 1 = Germany), and their interaction (time × country). A statistically significant interaction term (time × country) would suggest a differential change in the outcome in Germany relative to Austria, potentially attributable to the legalisation. Statistical significance was set at α = 0·05 (two-tailed), consistent with a non-directional hypothesis (H2) expecting no change in prevalence. No post-stratification weights were applied, as the weights were designed for population-level representativeness rather than for cannabis user subgroups. To mitigate possible confounding from changes in sampling over time, sociodemographic covariates showing significant associations with DUIC in baseline bivariate analyses (see Supplement S6) were included in the main model.

As sensitivity analyses, we estimated (1) a model without covariates, (2) a model including both sociodemographic and behavioural covariates, (3) a model using past 30-day DUIC as the outcome (to reduce potential recall bias and ensure that the recall period at t_1_ does not overlap with the pre-legalisation period), (4) a negative control model using past 12-month physical activity as the outcome (for rationale and negative control selection, see Supplement S9), and (5) a model replicating the main DUIC analysis in the total population (Sample 1). As DUIC was not assessed among less than monthly cannabis users in Germany at t_0_, values for this subgroup were imputed from the corresponding t_1_ data (for details, see note to Table S9.3). This approach assumes no change in DUIC in this group and results from this model should be interpreted with caution.

We reported aORs; for unadjusted ORs, see Supplement S9.

### DUIC episodes by cannabis use frequency and involvement of alcohol or other drugs

To estimate the relative burden of DUIC episodes by cannabis use frequencies and by the involvement of cannabis in combination with alcohol or other drugs, we conducted descriptive subgroup analyses restricted to Sample 3 (past year cannabis users who reported DUIC in the past 30 days, without medical-only users with a prescription, only t_1,_ pooled across countries). Total counts of DUIC episodes, as well as of estimated DUIC(−) and DUIC(+) episodes, were summarised across cannabis use frequency categories. We then calculated the proportional contribution of each group to the overall DUIC burden, separately for DUIC(−) and DUIC(+) episodes. Confidence intervals were derived via nonparametric bootstrapping (2000 resamples; R package *boot* v1·3–30).^28^ As sensitivity analyses, the same procedure was repeated using German data only, and using alternative assumptions for classifying DUIC episodes as DUIC(−)/(+) (see Supplement S11).

### Ethics approval

Ethical approval was obtained from the Local Psychological Ethics Committee of the Centre for Psychosocial Medicine in Hamburg, Germany (reference number: 0686). Participants’ consent for data use was obtained electronically before participation.

### Role of the funding source

The funder reviewed and approved the manuscript prior to submission and thus contributed to the decision to publish. AKB, employed by the funder, contributed as a co-author through methodology and manuscript review and editing. Beyond this, the funder had no role in the design of the study, data collection, analysis, interpretation, or manuscript writing.

## Results

### Study population

**Sample 1** included *n* = 6670 (t_0_) and *n* = 9692 (t_1_) respondents in Germany, and *n* = 2132 (t_0_) and *n* = 2102 (t_1_) in Austria (Table 1). Across timepoints and countries, gender distribution was approximately balanced (51% male; weighted and pooled) and mean age ranged from 42·3 years (SD = 13·6; Austria, t_0_; weighted) to 43·1 years (SD = 13·3; Germany, t_1_; weighted). Most participants in the German samples resided in urban or suburban areas, whereas urbanisation was more evenly distributed in Austria, with a higher share of rural residents. The majority had at least medium-level education. Sample characteristics remained stable across both waves. Post-stratification weights ranged from 0·42 (Germany, t_0_) to 3·4 (Austria, t_1_). The sample covered municipalities across the entire federal territories, with stable coverage between timepoints (Figure S3). The German sample was skewed towards higher socioeconomic status than the general population (Table S3.1; data only for Germany, as no equivalent Austrian socioeconomic index is available).

**Table 1:** Sample 1 (cannabis use) description.

	Germany					Austria				
	t_0_		t_1_		χ^2^ (df), *p*-value	t_0_		t_1_		χ^2^ (df), *p*-value
	Unweighted n (%)	Weighted n (%)	Unweighted n (%)	Weighted n (%)		Unweigthed n (%)	Weighted n (%)	Unweigthed n (%)	Weighted n (%)	
Proportion of men	3159 (47·4)	3375 (50·6)	4011 (41·4)	4915 (50·7)	0·02 (1), 0·901	1028 (48·2)	1076 (50·5)	946 (45·0)	1060 (50·5)	<0·01 (1), 0·988
Education level					6·35 (2), 0·050					2·05 (2), 0·388
Low	316 (4·7)	268 (4·0)	511 (5·3)	410 (4·2)		139 (6·5)	145 (6·8)	149 (7·1)	165 (7·8)	
Medium	3845 (57·6)	3711 (55·6)	6281 (64·8)	5558 (57·4)		1390 (65·2)	1399 (65·6)	1312 (62·4)	1380 (65·7)	
High	2503 (37·5)	2686 (40·3)	2888 (29·8)	3714 (38·3)		596 (28·0)	582 (27·3)	636 (30·3)	552 (26·3)	
Age group					8·41 (3), 0·076					7·08 (3), 0·101
18–24	752 (11·3)	681 (10·2)	651 (6·7)	861 (8·9)		192 (9·0)	211 (9·9)	244 (11·6)	260 (12·4)	
25–34	1542 (23·1)	1449 (21·7)	1652 (17·0)	2136 (22·0)		440 (20·6)	474 (22·2)	407 (19·4)	444 (21·1)	
35–44	1468 (22·0)	1479 (22·2)	2075 (21·4)	2144 (22·1)		469 (22·0)	461 (21·6)	465 (22·1)	461 (21·9)	
45–64	2908 (43·6)	3062 (45·9)	5314 (54·8)	4551 (47·0)		1031 (48·4)	986 (46·2)	986 (46·9)	937 (44·6)	
Degree of urbanisation					0·06 (2), 0·97					3·34 (2),·0·212
City	2925 (43·9)	2850 (42·7)	3909 (40·3)	4149 (42·8)		630 (29·4)	663 (31·1)	640 (31·3)	685 (32·6)	
Town/Suburb	2641 (39·6)	2704 (40·6)	3978 (41·0)	3913 (40·4)		764 (33·9)	718 (33·7)	741 (31·8)	654 (31·1)	
Rural	1100 (16·5)	1112 (16·7)	1801 (18·6)	1625 (16·8)		737 (36·6)	750 (35·2)	721 (36·9)	764 (36·3)	

Note. Missing data (≤0·3%) cause education and urbanisation sums <100%. Chi-square tests, with Rao-Scott correction, were applied to weighted counts to examine differences in sociodemographic variables between t_0_ and t_1_ within each country. Germany: *n*_t0_ = 6670, *n*_t1_ = 9692, Austria: *n*_t0_ = 2132, *n*_t1_ = 2102. t_0_: Nov–Dec 2023, t_1_: Nov 2024–Jan 2025.

**Sample 2** (at least monthly cannabis users without medical exemption; Table 2) included *n* = 393 (t_0_) and *n* = 589 (t_1_) respondents in Germany, and *n* = 86 (t_0_) and *n* = 92 (t_1_) in Austria. It skewed younger and more male, reflecting the demographic profile of cannabis users. Cannabis use frequency was relatively evenly distributed across monthly, weekly, and (almost) daily use, and remained stable over both time points, with a slight increase in monthly use observed in Austria at t_1_ and in (almost) daily use observed in Germany at t_1_. At t_1_, the proportion of participants holding a driver's licence was lower in both countries.

**Table 2:** Sample 2 (DUIC) and Sample 3 (DUIC episodes by polysubstance use) description.

	Sample 2				Sample 3
	Germany		Austria		Germany/Austria
	t_0_	t_1_	t_0_	t_1_	t_1_
Proportion of men, n (%)	267 (67·9)	344 (58·4)	50 (58·1)	57 (62·0)	57 (67·9)
Education level, n (%)					
Low	26 (6·6)	49 (8·3)	6 (7·0)	12 (13·0)	3 (3·6)
Medium	175 (44·5)	318 (54·0)	51 (59·3)	54 (58·7)	47 (56·0)
High	191 (48·6)	221 (37·5)	29 (33·7)	26 (28·3)	34 (40·5)
Age group, n (%)					
18–24	64 (16·3)	69 (11·7)	17 (19·8)	17 (18·5)	13 (15·5)
25–34	145 (36·9)	212 (36·0)	35 (40·7)	30 (32·6)	34 (40·5)
35–44	107 (27·2)	160 (27·2)	18 (20·9)	24 (26·1)	20 (23·8)
45–64	77 (19·6)	148 (25·1)	16 (18·6)	21 (22·8)	17 (20·2)
Degree of urbanisation, n (%)					
City	221 (56·2)	302 (51·3)	34 (39·5)	35 (38·0)	40 (47·6)
Town/Suburb	126 (32·1)	205 (34·8)	30 (34·9)	37 (40·2)	28 (33·3)
Rural	46 (11·7)	81 (13·8)	22 (25·6)	20 (21·7)	16 (19·0)
Cannabis use frequency, n (%)					
Less than monthly	–	–	–	–	14 (16·7)
Monthly	145 (36·9)	200 (34·0)	29 (33·7)	35 (38·0)	20 (23·8)
Weekly	133 (33·8)	190 (32·3)	23 (26·7)	23 (25·0)	21 (25·0)
Daily	115 (29·3)	199 (33·8)	34 (39·5)	34 (37·0)	29 (34·5)
Proportion with driver's licence, n (%)	310 (78·9)	445 (75·6)	63 (73·3)	63 (68·5)	76 (90·5)

Note. Unweighted values. Sample 2: Missing data (≤0·3%) cause education and urbanisation sums <100%. Germany: *n*_t0_ = 393, *n*_t1_ = 589, Austria: *n*_t0_ = 86, *n*_t1_ = 92. Sample 2 includes at least monthly cannabis users without medical exemption (respondents with exclusive medical use and a prescription were excluded). Sample 3: *n*_t1, GER + AT_ = 84. Sample 3 includes past-year cannabis users without medical exemption reporting DUIC in the past 30 days. t_0_: Nov–Dec 2023, t_1_: Nov 2024–Jan 2025.

**Sample 3** (past-year cannabis users without medical exemption reporting DUIC in the past 30 days; t_1_ only; Table 2) included *n* = 84 respondents and was slightly younger on average and exhibited a modestly higher proportion of male respondents compared to Sample 2. 34·5% reported using cannabis on a (near) daily basis which is similar to Sample 2.

### Changes in cannabis use (H1) and DUIC (H2)

**Fig. 2:**
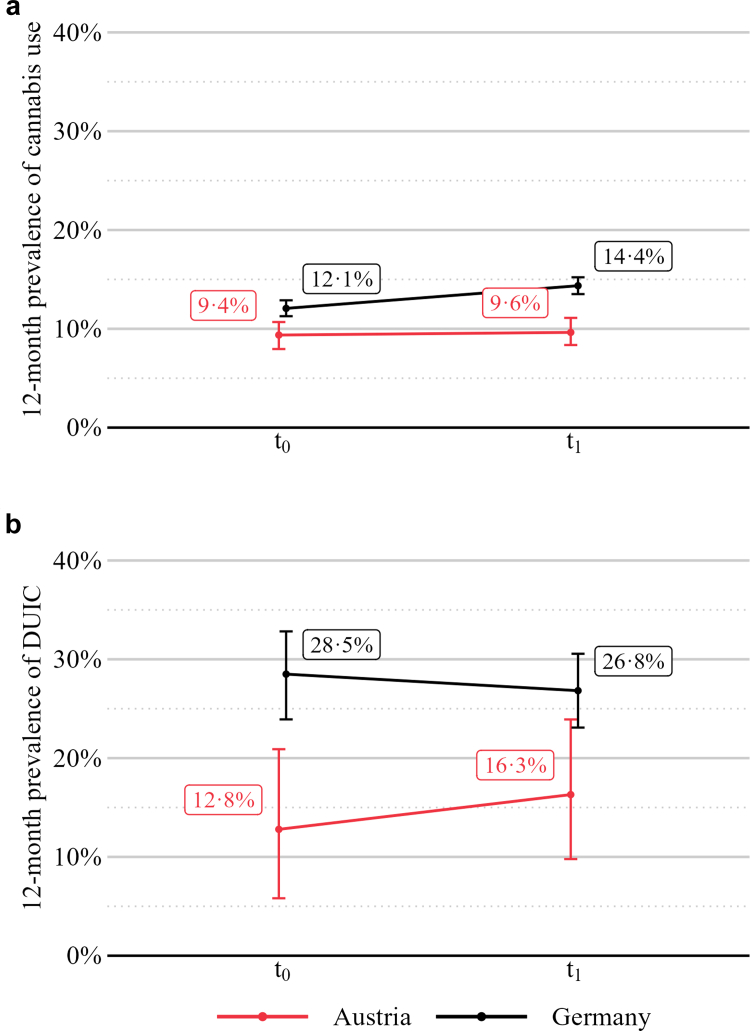
**12-month prevalence of cannabis use (a) and driving under the influence of cannabis (b) among adults in Germany and Austria, before and after legalisation.** Panel a:weighted estimates based on Sample 1 of the general population aged 18–64 years. Sample sizes: Germany: *n*_t0_ = 6670, *n*_t1_ = 9692, Austria: *n*_t0_ = 2132, *n*_t1_ = 2102. Panel b: unweighted estimates based on Sample 2 of at least monthly cannabis users without a medical exemption. Sample sizes: *n*_t0_ = 393, *n*_t1_ = 589, Austria: *n*_t0_ = 86, *n*_t1_ = 92 Error bars represent 95% bootstrap confidence intervals. t_0_: Nov–Dec 2023, t_1_: Nov 2024–Jan 2025.

**Cannabis use (H1):** Prior to legalisation (t_0_), the 12-month prevalence of cannabis use was 12·1% (95% *CI* 11·3–12·9) in Germany and 9·4% (95% *CI* 8·0–10·7) in Austria. Following legalisation (t_1_), prevalence increased to 14·4% (95% *CI* 13·5–15·2) in Germany and 9·6% (95% *CI* 8·4–11·1) in Austria (Fig. 2a). The interaction term between time (t_1_) and country (Germany) was not statistically significant (*OR* = 1·18, 95% *CI* 0·94–1·48, *p* = 0·141; weighted model, see Table 3 for full regression results), indicating no evidence of a differential change in 12-month cannabis use prevalence between Germany and Austria after legalisation. The minimum detectable DiD-effect at 80% power was OR = 1·40, smaller true effects cannot be ruled out (see Table S7).

**Table 3:** Results from logistic regression model (difference-in-differences) for past 12-month cannabis use (Hypothesis 1).

	*OR* (95% CI)	*p*-value
**Primary analysis 1: past 12-month cannabis use**		
*Time*: t_1_ (reference: t_0_)	1·03 (0·84–1·27)	0·768
*Country*: Germany (reference: Austria)	1·33 (1·13–1·57)	**<0·001**
t_1_ × Germany (DiD-effect)	1·18 (0·95–1·48)	0·141

Note. DiD, difference-in-differences. Odds Ratios (OR) and 95% Confidence Intervals (CI) are reported from binomial logistic regression models using post-stratification weights. DiD, difference-in-differences. Sample sizes (Sample 1): Germany: *n*_t0_ = 6670; *n*_t1_ = 9692; Austria: *n*_t0_ = 2132; *n*_t1_ = 2102. t_0_: Nov–Dec 2023, t_1_: Nov 2024–Jan 2025. R^2^ = 0·006 (Nagelkerke); χ^2^(3) = 67·9, *p*<0·001. Bold values indicate statistical significance at *p* < 0·05. For unweighted or adjusted models, see Table S9.1.

Sensitivity analyses (unweighted; unweighted including sociodemographic covariates: age group, gender, education level, degree of urbanisation, see Table S6.1 for variable selection; unweighted including both sociodemographic and behavioural covariates: frequency of alcohol use, gambling and physical activity, see Table 6.2 for variable selection; negative control model) yielded consistent results. However, without controlling for Austria, cannabis use in Germany post-legalisation increased significantly (*OR* = 1·22, 95% *CI* 1·11–1·34, *p* < 0·001, weighted model; see Table S9.1 for sensitivity analyses results).

**DUIC (H2):** Prior to legalisation (t_0_), the 12-month prevalence of DUIC was 28·5% (95% *CI* 23·9–32·8) in Germany and 12·8% (95% *CI* 7·0–19·8) in Austria among at least monthly cannabis users (excluding medical-only users with a prescription; see Supplement S10 for DUIC prevalence in this subgroup). Following legalisation (t_1_), prevalence decreased to 26·8% (95% *CI* 23·3–30·6) in Germany and increased to 16·3% (95% *CI* 8·7–25·0) in Austria (Fig. 2b). The main adjusted model controlled for age group, gender, education level, and driver's licence status—sociodemographic variables found to be significantly associated with DUIC in baseline bivariate analyses (Table S6.1). The interaction term between time (t_1_) and country (Germany) was not statistically significant (*p* = 0·408, *aOR* = 0·68, 95% *CI* 0·27–1·68; see Table 4 for full regression results), indicating no evidence of a differential change in 12-month DUIC prevalence between Germany and Austria after legalisation. The minimum detectable DiD-effect at 80% power was OR = 3·68 (see Table S7).

**Table 4:** Results from logistic regression model (difference-in-differences) for past 12-month DUIC (Hypothesis 2).

	*aOR* (95% CI)	*p*-value
**Primary analysis 2: past 12-month DUIC**		
*Time*: t_1_ (reference: t_0_)	1·58 (0·67–3·82)	0·295
*Country*: Germany (reference: Austria)	2·56 (1·34–5·33)	**0·007**
t_1_ × Germany (DiD-effect)	0·68 (0·27–1·68)	0·408
*Gender*: female (reference: male)	0·68 (0·50–0·91)	**0·010**
*Driver's licence*: yes (reference: no)	2·10 (1·43–3·14)	**<0·001**
*Age group* (reference: 18–24 years):		
25–34 years	1·14 (0·75–1·76)	0·488
35–44 years	0·76 (0·49–1·21)	0·185
45–64 years	0·45 (0·27–0·74)	**<0·001**
*Education* (reference: low):		
Medium	2·10 (1·06–4·68)	**0·048**
High	3·20 (1·59–7·18)	**0·002**

Note. DiD, difference-in-differences; DUIC, driving under the influence of cannabis, t_0_: Nov–Dec 2023, t_1_: Nov 2024–Jan 2025. Sample sizes (Sample 2, at least monthly cannabis users without medical exemption): Germany: *n*_t0_ = 393, *n*_t1_ = 589, Austria: *n*_t0_ = 86, *n*_t1_ = 92. R^2^ = 0·115 (Nagelkerke); χ^2^(0) = 94·0, *p* < 0·001. All generalised variance inflation factors were below 1·13, except for *Time, Country* and the interaction *Country*x*Time*, which is expected as interactions are inherently correlated with the main effects. Bold values indicate statistical significance at *p*< 0·05. For unadjusted OR, see Table S9.2.

Sensitivity analyses (unadjusted; adjusted for both sociodemographic and behavioural covariates: frequency of alcohol use and gambling, see Table S6.1 for variable selection; past 30-day DUIC; negative control model; DUIC among the total population) consistently showed a non-significant DiD-effect (Tables S9.2 and S9.3).

DUIC was modestly negatively associated with NQ– (*OR* = 0·60, 95% *CI* 0·48–0·74, *p* < 0·001), with no significant interaction over time (*OR* = 1·12, 95% *CI* 0·87–1·46, *p* = 0·38). No significant changes were observed in self- (*p* = 0·936; χ^2^ (2) = 0·13) or perceived (*p* = 0·302; χ^2^ (2) = 2·39) stigmatisation toward people using cannabis between t_0_ and t_1_ (Sample 2, Germany only; for details, see Supplement S5), indicating stable social desirability post-legalisation.

#### DUIC episodes by cannabis use frequency and involvement of alcohol or other drugs

**Fig. 3:**
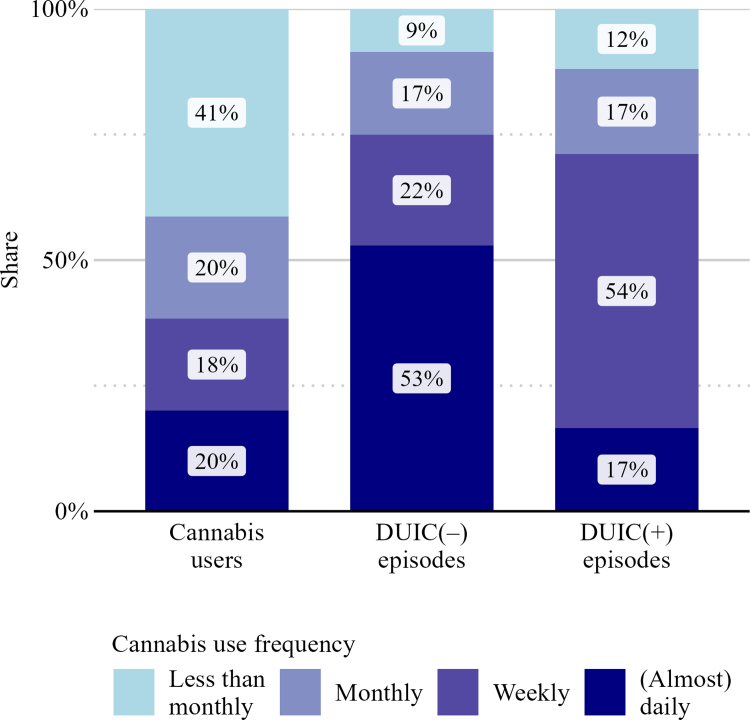
**Distribution of cannabis users, DUIC(−) episodes, and DUIC(+) episodes by cannabis use frequency.** DUIC(−) = driving under the influence of cannabis only, DUIC(+) = driving under the influence of cannabis in combination with alcohol or other drugs. *n* past year cannabis users = 1160 (excluding pure medical users with a prescription), *n* DUIC(−) episodes = 392, *n* DUIC(+) episodes = 108, from *n* = 84 respondents (Sample 3, post-legalisation, German and Austrian data are pooled).

Overall, respondents (*n* = 84, Germany and Austria pooled) in Sample 3 reported an estimated 500 DUIC episodes in the past 30 days (median = 2·5; *IQR* = 1·6–6·5; maximum = 60). Of all reported DUIC episodes, 392 (78·5%, 95% *CI* 63·3–90·4) were classified as DUIC(−), while 108 (21·5%, 95% *CI* 9·6–36·7) were classified as DUIC(+). Fig. 3 displays the contribution of cannabis use frequency groups to the DUIC burden, distinguishing DUIC(−) and DUIC(+) episodes, and their respective proportions in the cannabis-using population. Daily users constituted only 20% (95% *CI* 17·8–22·5) of the cannabis-using population but accounted for 52·9% (95% *CI* 32·5–69·1) of all DUIC(−) episodes and 16·6% (95% *CI* 3·0–44·3) of DUIC(+) episodes. In contrast, weekly users made up 18·4% (95% *CI* 16·1–20·6) of users and accounted for 22·0% (95% CI 11·1–36·8) of DUIC(−) episodes, but contributed 54·5% (95% *CI* 8·2–80·3) of DUIC(+) episodes. Meanwhile, individuals who used cannabis less than monthly represented the largest share of users (41·3%, 95% *CI* 38·4–44·1) and contributed 8·6% (95% *CI* 3·7–15·9) of DUIC(−) and 12·0% (95% *CI* 3·0–36·6) of DUIC(+) episodes. Sensitivity analyses restricted to German respondents (Figure S11.1), as well as those applying alternative assumptions for classifying DUIC episodes as DUIC(−)/(+) (Figures S11.2 and S11.3), confirmed the overall pattern, despite some minor variation in the magnitude of estimates.

## Discussion

This study is the first to evaluate the short-term impact of cannabis legalisation in Germany on both cannabis use and DUIC. Employing a DiD study design with Austria as control, the findings suggest that cannabis use further increased in Germany, however, this trend is comparable to what can be observed in Austria, where cannabis possession and cultivation remained illegal. Moreover, DUIC among at least monthly cannabis users appears not to have increased in Germany in the first months after the liberalisation of cannabis laws for personal use and for traffic participation.

Commercial cannabis legalisation in North America has been linked to small increases in use prevalence (recent overview:^4^), particularly in jurisdictions with more liberal policies,^29^ driven by, for instance, increased availability of legal cannabis.^30^ In Germany, however, commercial sales of recreational cannabis remain prohibited and only a few dozen ‘cannabis clubs' had been authorised, covering less than 1% of estimated users at the time of the survey. Meanwhile, the rapidly expanding medical market—imports of medical cannabis flower nearly quadrupled in 2024^31^—may partially offset restricted availability. It remains to be investigated how the availability of medical and recreational cannabis in Germany affects cannabis use prevalence. So far, the non-significant increase in past-year use prevalence observed in this and another survey^32^ suggest that prior trends^9^ remain unchanged by the Cannabis Act.

Official crash statistics show a continued rise in MVCs under the influence of psychoactive substances (including but not limited to cannabis), with no abrupt change following legalisation.^33^ Our findings are consistent with this trend: population-level DUIC is believed to increase with rising cannabis use prevalence at stable DUIC rates among users. As this trend was also observed in Austria, no short-term changes appear to have been caused by cannabis legalisation. While both MVC statistics and survey data suggest that pre-existing trends have continued, two caveats in the data should be considered. First, MVC statistics do not allow a quantification of the cannabis-specific contribution. Second, the population-level DUIC estimate (Table S9.3) relies on the assumption that DUIC among less than monthly users remained unchanged; violations of this assumption would bias the estimate. Embedding this finding in previous research,^4^ we can summarise that cannabis legalisation is not necessarily causing a short-term increase in DUIC. Nevertheless, long-term monitoring is necessary to determine whether the legalisation of cannabis will gradually impact DUIC and traffic safety. Importantly, the implications of exemptions for cannabis patients to drive after using medical cannabis also warrant further study.

Previous findings of increased cannabis involvement in fatal crashes after legalisation^5^ or in regions with more liberal cannabis policies^6^ may be explained by higher prevalence of use in the population rather than changes in driving behaviour among users in response to legalisation. In other words, population-level increases in cannabis use may result in an increase in the number of MVCs—despite stable DUIC patterns among users. This aligns with Canadian survey data, where DUIC increased among all respondents but decreased moderately among cannabis users five years after legalisation.^14^ Like Germany, Canada applies a per-se THC limit (2 ng/ml whole blood, ∼2·8 ng/ml blood serum), with exceedance constituting a summary conviction offence (Blood Drug Concentration Regulations, SOR/2018-148). Among users, our data did not indicate short-term behavioural adaptation in DUIC following the introduction of the new THC-limit for most adult drivers (from 1 ng/ml to 3·5 ng/ml blood serum, the 1 ng/ml limit for novice drivers, those under 21, and combined use with alcohol remained unchanged). Such behavioural adaptation may be moderated by enhanced deterrence^12^ or prevention measures. In Germany, the legalisation coincided with prevention campaigns (e.g., “Don't Drive High”) and announcements of intensified roadside controls by police authorities in several federal states; however, these factors were not examined in the present study. Similar to previous studies, we cannot ascertain if self-reported DUIC was consistent with the THC-limit, as no objective blood measurements were available. Subtler behavioural adjustments—such as shorter waiting times before driving—would not be captured by our DUIC measure, but may be detectable with THC blood concentrations. A regionally limited study from southwest Saxony using administrative police data from roadside drug testing observed higher THC blood concentrations following legalisation^34^; however, the absence of information on testing strategies limits the interpretability of these findings. Therefore, more robust evidence is needed from studies examining changes in THC levels in relation to cannabis legalisation.

Findings of our explorative analysis suggest that the majority of DUIC episodes involved only cannabis and no alcohol or other drugs. Most studies on this subject have not addressed co-use of alcohol or other drugs in the context of DUIC, despite elevated risks and differential fines for mixing cannabis and other substances (e.g., Canada: Blood Drug Concentration Regulations, SOR/2018-148). DUIC(−) episodes were concentrated among daily users, whereas DUIC(+) episodes were concentrated among weekly users. While our study does not provide evidence on impairment by use frequency, previous research suggests that daily users may experience less impairment from the same dose of THC due to tolerance.^16^ Therefore, the greatest potential traffic safety risk among cannabis users may occur in those using cannabis weekly and combine it with other substances before driving. Further research is required to assess the implications for traffic safety. Identifying a key research gap, we propose that future studies account for the co-use of alcohol or other drugs in the context of DUIC. This differentiation may be crucial to deepen our understanding on the impact of cannabis legalisation on traffic safety.

Certain limitations should be acknowledged. We relied on quota samples from ISO-certified online access panels, that may not be representative of the general populations of Germany and Austria. While representativeness is not necessarily required for testing of the hypotheses, generalisability of the findings is limited to the recruited samples. Notably, the sample is skewed towards individuals with higher socioeconomic status while hard-to-reach populations such as people experiencing homelessness, people in institutions, or without internet access are not sufficiently covered and may exhibit other trends in cannabis use and DUIC. Comparisons with national probability-based surveys showed substantial overlap, though addictive behaviours were higher in our samples (Table S3.2). True population prevalence may not be captured; however, higher prevalence in our samples may reflect reduced underreporting, supporting the suitability for studying sensitive behaviours like cannabis use and DUIC. Gender- or ethnicity-stratified analyses were not conducted, as the primary focus of this study was on population-level effects of legalisation. Future research is warranted to assess potential differential effects.

DUIC was assessed via self-report, and no objective verifications (e.g., THC levels or tests of driving impairment) were available. Therefore, DUIC may be affected by recall and social desirability biases. However, social desirability regarding DUIC in our sample remained stable over time, making substantial time-varying bias unlikely.

As a quasi-experiment, unmeasured confounding cannot be fully excluded. Further, spillover to Austria or anticipation effects are possible; although at baseline (Nov–Dec 2023), the implementation of legalisation was still uncertain (parliament passed the bill only in Feb 2024), likely limiting behaviour changes driven by expectations of legalisation.

The t_1_-survey assessed 12-month DUIC only 8 months after legalisation, so the reference period post-legalisation overlaps with the pre-period, which may lead to an underestimation of the true legalisation effect. However, a sensitivity analysis using past-30-day DUIC confirmed the absence of a significant change, suggesting the overlapping periods do not explain the null finding. While cannabis policy changes can have both immediate and lagged effects,^35^ the present study, given its short follow-up period since legalisation, captures only immediate effects on cannabis use and DUIC.

Testing the parallel trends assumption underlying the DiD was limited by the absence of earlier survey waves. Using pre-legalisation data from national surveys and official statistics, we found no evidence of divergent pre-trends in cannabis use or drug-impaired MVCs between Germany and Austria. Although constrained by the lack of Austrian cannabis use data after 2020 and of cannabis-specific MVC data, these findings, together with the absence of major policy or enforcement changes and the overall structural comparability between both countries, support the plausibility of the parallel trends assumption.

The sample sizes for at least monthly cannabis users (Sample 2, *n* = 86–92 in Austria and *n* = 393–589 in Germany per timepoint) were sufficient to detect main effects reflecting temporal changes in DUIC within Germany. However, the smaller Austrian sample limited the power to detect interaction effects (minimum detectable DiD-effect: OR = 3·68), meaning small to medium between-country differences may go undetected. Nonetheless, the observed decline in DUIC in Germany and increase in Austria support the hypothesis of non-increasing DUIC prevalence among at least monthly users, despite limited power.

Overall, this study found no short-term impact on cannabis use or DUIC after legalisation of cannabis in Germany. Exploratory analyses indicate that daily users reported the most DUIC episodes involving cannabis only, whereas weekly users reported the most DUIC episodes involving cannabis in combination with other substances. Further studies with longer observation periods and other data, most importantly MVC involving substance use and toxicological information, are required to monitor the long-term implications of the law.

## Contributors

AS: conceptualization, methodology, formal analysis, data curation, writing–original draft, visualization; AKB: methodology, writing–review & editing; MR: methodology, writing–review & editing; UV: methodology, writing–review & editing, funding acquisition; JM: conceptualization, data verification, funding acquisition, methodology, project administration, supervision, writing—review & editing. All authors had full access to the data in the study and accept responsibility to submit for publication.

## Data sharing statement

Underlying data are not publicly available. The statistical code is available on GitHub (https://github.com/annasrz/CannaStreet-DUICLeg).

## Editor note

The Lancet Group takes a neutral position with respect to territorial claims in published maps and institutional affiliations.

## Declaration of interests

AS received presentation honoraria from the Bayerische Akademie für Sucht-und Gesundheitsfragen and conference travel expenses from the Deutsche AIDS-Hilfe e.V. Unrelated to the present work, JM worked as consultant for and received honoraria from various public health organisations (World Health Organization, European Monitoring Centre for Drugs and Drug Addiction, national non-governmental organisations) and received payment for expert testimony in the German parliament. AKB was a member of the expert commission on the legal THC threshold and is employed at the Federal Highway and Transport Research Institute. MR received presentation honoraria from the Villa Schöpflin–Zentrum für Suchtprävention and reimbursement of travel expenses from the Klinikum Itzehoe–Zentrum für Psychosoziale Medizin. UV worked as consultant for and received honoraria from Hexal AG. JM, UV, MR and AS are involved in a research project to analyse the effects of the cannabis legalisation in Germany funded by the German Ministry of Health.

## Appendix B Supplementary data

Supplement

German Translation
