# Supplementary material for: Short-term effects of cannabis legalisation in Germany on driving under the influence of cannabis: a difference-in-differences analysis using Austria as a control
Source: Lancet Reg Health Eur. 2026 Jan 23;63:101593. doi: 10.1016/j.lanepe.2026.101593 (PMC12860694; doi:10.1016/j.lanepe.2026.101593)
Supplement: Supplement [file mmc1.docx]

**Supplementary material**

**Title:** Short-term effects of cannabis legalisation in Germany on driving under the influence of cannabis: A difference-in-differences analysis using Austria as a control

**Brief title:** Cannabis Legalisation and DUIC in Germany

**Authors**: Anna Schranz, Anja Knoche-Becker, Moritz Rosenkranz, Uwe Verthein, Jakob Manthey

Table of contents

[Supplement S1: Parallel trends assumption 2](#_Toc217058613)

[S1.1 Cannabis Use 2](#_Toc217058614)

[S1.2 DUIC 3](#_Toc217058615)

[Supplement S2: Survey metrics 5](#_Toc217058616)

[Supplement S3: Sample descriptions 6](#_Toc217058617)

[Supplement S4: Questionnaire items used in analyses 8](#_Toc217058618)

[Supplement S5: Social desirability bias in measuring DUIC 10](#_Toc217058619)

[Supplement S6: Selection of covariates for DiD models 12](#_Toc217058620)

[Supplement S7: Minimum detectable effect 14](#_Toc217058621)

[Supplement S8: Sample weighting 15](#_Toc217058622)

[Supplement S9: Sensitivity analyses for DiD models 16](#_Toc217058623)

[Supplement S10: DUIC among at least monthly medical-only cannabis users with a prescription 21](#_Toc217058624)

[Supplement S11: Sensitivity analyses for exploratory analysis: DUIC episodes by cannabis use frequency and involvement of alcohol or other drugs 22](#_Toc217058625)

[References 25](#_Toc217058626)

# **Supplement S1: Parallel trends assumption**

The validity of the DiD framework relies on the assumption that, in the absence of legalisation, trends in cannabis use and DUIC in Germany would have followed a parallel trajectory to those in Austria. Establishing pre-legalisation parallel trends is essential to attribute post-legalisation changes to the legalisation rather than to pre-existing national differences in trends. As no further pre-legalisation waves than t_0_ of our own survey were available, we evaluated this assumption using external pre-2024 data.

## S1.1 Cannabis Use

For adult cannabis use, we analysed aggregated national survey data from Germany (Epidemiological Survey of Substance Abuse, ESA; 2009–2021) and Austria (Gesundheit Österreich GmbH, GÖG; 2008–2020, see Figure S1.1). For each survey year, we estimated the number of cannabis users (n) based on reported 12-month prevalence and sample size (N), and fitted a logistic regression model with a binomial denominator (n of N) as the outcome, including *year* (continuous), *country*, and their interaction as predictors. The interaction was non-significant, suggesting comparable pre-legalisation trends in both countries (see Table S1.1). However, this has to be interpreted with caution given the small number of country-year observations (n=8).

Visually consistent trends were also observed in adolescent cannabis use (European School Survey Project on Alcohol and Other Drugs; ESPAD^1^, see Figure S1.1), further supporting the plausibility of the parallel trends assumption.

**Figure S1.1. Trends in past 12-month cannabis use prevalence in Germany and Austria among adults and adolescents, 2003–2024 (ESA, ESPAD, GOEG) to assess parallel trends assumption for DiD analyses**Data for adults (aged 18–64) in Germany are from the Epidemiological Survey of Substance Abuse (ESA^2,3^); data for adults (aged 15–64) in Austria are from Gesundheit Österreich GmbH (GÖG, retrieved via EUDA^4^). Data for adolescents (aged 15–16) are from the European School Survey Project on Alcohol and Other Drugs (ESPAD^1^). Data from ESA 2024 and ESPAD were not included in the statistical trend analysis.
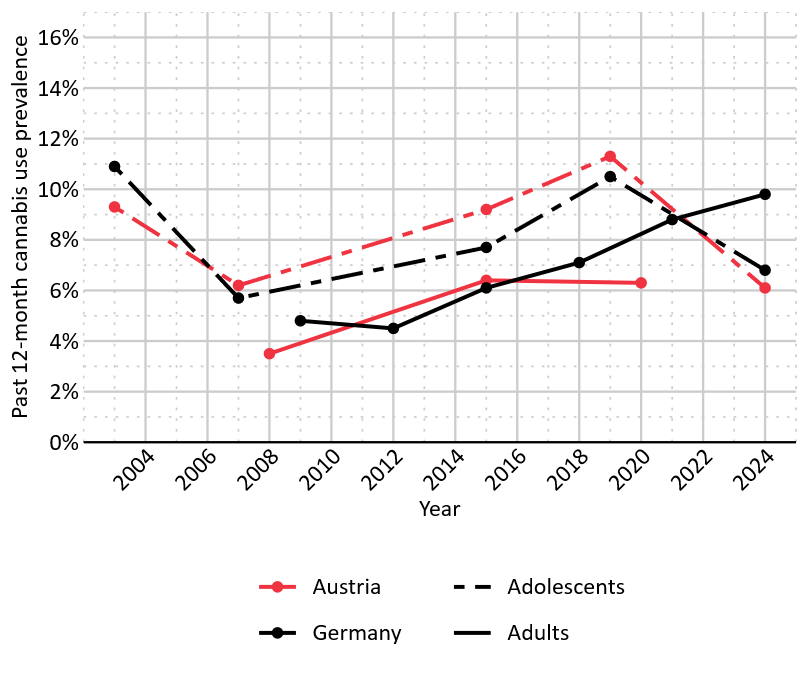


**Table S1.1. Logistic regression predicting pre-legalisation trends in past 12-month cannabis use (Germany vs. Austria, 2008–2021) based on aggregated ESA and GÖG survey data**

|  | ***OR* (95% CI)** | ***p*-value** |
| --- | --- | --- |
| **Outcome: Past 12-month cannabis use (n/N)** | |  |
| *Year* | 1·05 (1·03–1·07) | **<·001** |
| *Country*: Germany (reference: Austria) | 1·03 (0·87–1·23) | ·727 |
| *Year* x *Country* | 1·01 (0·99–1·03) | ·157 |

Note. OR=odds ratio; CI=confidence interval. Interaction term tests whether pre-legalisation trends differed between countries. Year was centered at the earliest year included in the analysis (i.e., 2008=0). Data for adults (aged 18–64) in Germany are from the Epidemiological Survey of Substance Abuse (ESA^2^); data for adults (aged 15–64) in Austria are from Gesundheit Österreich GmbH (GÖG, retrieved via EUDA^4^). *N*=8 country-year observations. Sample sizes per country-year ranged from *N*=8,030 to 9,204 in Germany and *N*=3,477 to 4,650 in Austria.

## S1.2 DUIC

DUIC is not routinely measured in surveys, and counts of cannabis-specific motor vehicle crashes (MVCs) are not reported in official crash statistics. Therefore, we used national counts of drug-impaired MVCs involving personal injury in Austria and persons involved in drug-related MVCs involving personal injury in Germany as a proxy indicator (see Figure S1.2). We fitted a Poisson regression model with the annual number of (persons involved in) drug-impaired MVCs as the outcome, including *year* (continuous), *country*, and their interaction as predictors. The log of the annual population sizes^5–7^ was included as an offset, modelling crash rates rather than absolute counts. The interaction was non-significant, suggesting comparable pre-legalisation trends in both countries (see Table S1.2).

**Figure S1.2.** Trends in drug-impaired motor vehicle crashes in Germany and Austria, 2019–2024 to assess parallel trends assumption for DiD analyses

Rate of persons involved in drug-impaired MVCs involving personal injury (Germany) and rate of drug-impaired MVCs involving personal injury (Austria) per 100,000 inhabitants. Crashes involving only alcohol are excluded. Data were obtained from the Federal Statistical Office of Germany^8^ and from Statistics Austria^9^. Population data^5–7^ were used to calculate rates. 2024 data were not included in the statistical trend analysis.


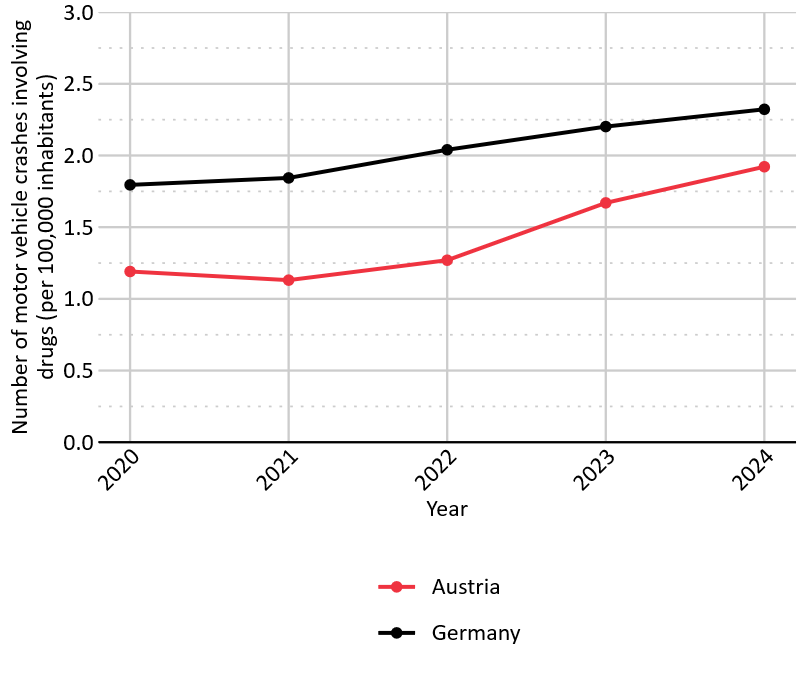


**Table S1.2. Poisson regression predicting pre-legalisation trends in annual drug-impaired motor vehicle crash rates (Germany vs. Austria, 2020–2023)**

|  | ***IRR* (95% CI)** | ***p*-value** |
| --- | --- | --- |
| **Outcome: annual rate of (persons involved) in drug-impaired MVCs** | |  |
| *Year* | 1·13 (1·04–1·22) | **·004** |
| *Country*: Germany (reference: Austria) | 1·62 (1·38–1·93) | **< ·001** |
| *Year* x *Country* | 0·95 (0·87–1·03) | ·255 |

Note. IRR=incident rate ratio; CI=confidence interval; MVC=motor vehicle crash. Interaction term tests whether pre-legalisation trends differed between countries. Log of population size was included as an offset. Year was centered at the earliest year included in the analysis (i.e., 2020=0). Dispersion=1·20. Data on drug-impaired MVCs and population size were obtained from the Federal Statistical Office of Germany^5,8^ and from Statistics Austria.^6,7,9^ Austria: rate of drug-impaired MVCs with personal injury; Germany: rate of persons involved in drug-related MVCs with personal injury.

# Supplement S2: Survey metrics

**Figure S2. Survey participation metrics and sample preprocessing.**

t₀: Nov–Dec 2023, t₁: Nov 2024–Jan 2025; GER = Germany, AT = Austria. ^1^: During coarse cleaning, cases with unrealistic response times (‘speeders’) and little response variability (‘straightliners’) were excluded. ^2^: Target samples were defined as individuals reporting at least monthly cannabis use for GER t_0_ and as any cannabis use in the past 12 months in GER t_1_, AT t_0_ and AT t_1_. ^3^: In the iterative cleaning step, cases were excluded from the target sample based on additional control questions and plausibility checks (the target sample received a more detailed questionnaire, allowing for finer-grained quality check). Cases were proportionally removed from the full sample to maintain a consistent ratio with the target sample and avoid systematic bias. Proportional exclusions from the full sample were based on data quality, prioritising short interviews and low-quality responses for exclusion. ^4^:To ensure sample independence, individuals participating in both t_0_ and t_1_ were excluded.
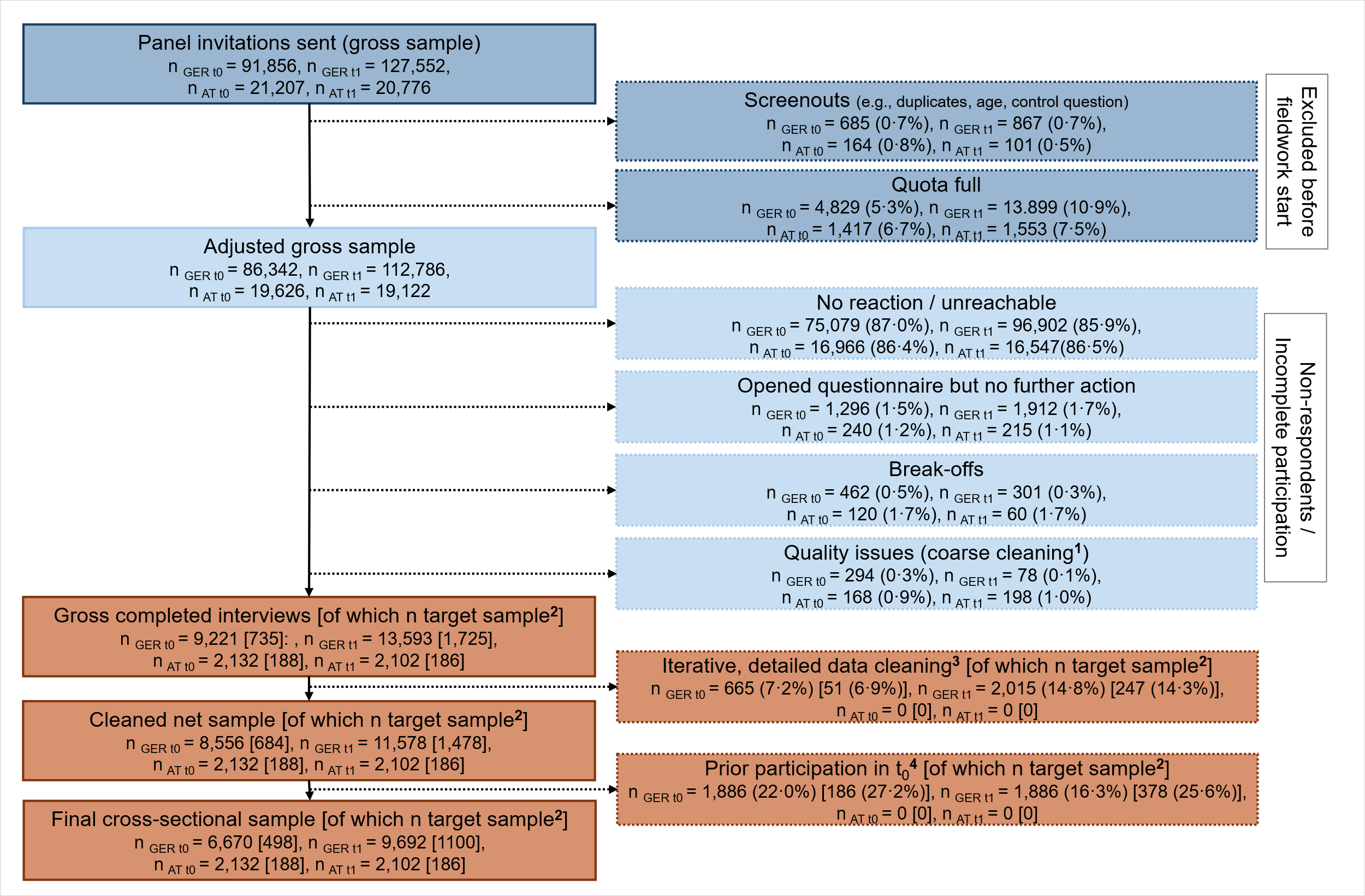


**Table S2. Recruitment channels of the ‘Bilendi’ panel**

| Channel | Share |
| --- | --- |
| Co-registration | 26% |
| Display | 17% |
| Affiliate Marketing | 14% |
| Bilendi App | 12% |
| Organic | 9% |
| Email Campaigns | 8% |
| Search Engine Advertising | 8% |
| Social Ads | 3% |
| Referral Programs | 3% |
| Total | 100% |

Note. >90% of panellists are recruited actively, i.e., through targeted measures. All panellists are recruited exclusively through permission-based recruitment techniques. River sampling is not employed by Bilendi.

# Supplement S3: Sample descriptions

**Figure S3. Geographic distribution of survey responses in Germany and Austria for Sample 1 (cannabis use), by country and study wave.**

t₀: Nov–Dec 2023, t₁: Nov 2024–Jan 2025. Maps show municipalities with at least one survey respondent. National borders are shown in black. Areas without respondents are not displayed. Sample sizes: Germany: *n*_t0_=6,670, *n*_t1_=9,692; Austria: *n*_t0_=2,132, *n*_t1_=2,102.

**
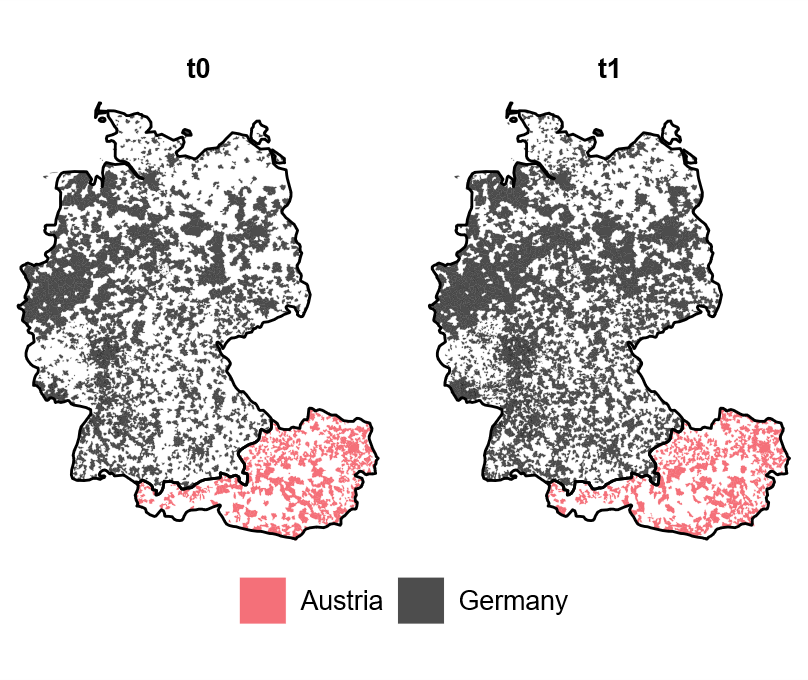
**

**Table S3.1. Socioeconomic Distribution of Sample 1 (cannabis use) based on the German Index of Socioeconomic Deprivation (GISD), by survey wave (Germany only).**

|  | | **Germany** | |
| --- | --- | --- | --- |
| **GISD Quintile** | **t_0_ (n, %)** | **t_1_ (n, %)** | **Population (%)** |
| 1 (lowest deprivation) | 2,004 (31·9) | 2,740 (31·3) | 20 |
| 2 | 1,335 (20·8) | 2,023·(21·4) | 20 |
| 3 | 1,277 (17·7) | 1,712 (17·2) | 20 |
| 4 | 1033 (14·3) | 1,481 (13·7) | 20 |
| 5 (highest deprivation) | 1041 (15·2) | 1,718 (16·2) | 20 |
| *missing* | 10 (0·1) | 18 (0·2) | - |

Note. Counts are unweighted; percentages are weighted. Socioeconomic deprivation was assessed using the German Index of Socioeconomic Deprivation (GISD), developed by the Robert Koch Institute (RKI).^10^ Sample sizes (Sample 1, Germany only): n_t0_= 6,670, n_t1_= 9,692. t₀: Nov–Dec 2023, t₁: Nov 2024–Jan 2025.

**Table S3.2. 12-month prevalence of cannabis, alcohol, tobacco use, and gambling in the German and Austrian Sample 1 (cannabis use) at t₀ and t₁, compared with other national population surveys**

| **Sample** | **Cannabis use** | **Alcohol use** | **Gambling** | **Tobacco use** |
| --- | --- | --- | --- | --- |
| Germany, t_0_ | 12·1%  [11·3–12·9] | 77·1%  [76·0–78·2] | 42·4%  [41·2–43·7] | 35·9%  [34·8–37·1] |
| Germany, t_1_ | 14·4%  [13·5–15·2] | 77·1%  [76·2–78·0] | 44·4%  [43·2–45·4] | 35·5%  [34·4–36·5] |
| Other German population surveys | 8·8%  ESA, 2021^2^ | 84·7%  ESA, 2021^11^ | 36·5%  Gambling Survey, 2023^12^ | 28·3%  DEBRA, 2024^13^ |
| Austria, t_0_ | 9·4%  [8·0–10·7] | 78·9%  [77·1–80·6] | 50·2%  [48·0–52·4] | 40·2%  [38·1–42·5] |
| Austria, t_1_ | 9·6%  [8·4–11·1] | 76·9%  [75·0–78·8] | 54·6%  [52·4–56·9] | 41·6%  [39·2–43·8] |
| Other Austrian population surveys | 6·3%  GPS GÖG, 2020^4^ | 81%  GPS GÖG, 2020^14^ | 47%  GPS GÖG, 2020^14^ | 24%  GPS GÖG, 2020^14^ |

Note. Values are weighted with 95% bootstrap confidence intervals shown in brackets. t₀: Nov–Dec 2023, t₁: Nov 2024–Jan 2025. Germany: n_t0_=6,670; n_t1_=9,692, Austria: n_t0_= 2,132, n_t1_=2,102. ESA=Epidemiological Survey of Substance Abuse; DEBRA=German Study on Tobacco Use; GPS=General Population Survey (Austria); GÖG=Gesundheit Österreich GmbH.

# Supplement S4: Questionnaire items used in analyses

**Table S4. Questionnaire items used in analyses (in alphabetical order)**

| **Item name** | **Item wording** | **Response scale** |
| --- | --- | --- |
| AGE | Please enter your age in years. | ___ years |
| DUIC | Have you ever driven a motor vehicle (e.g. car, motorbike) within 2 hours of using cannabis? | Yes, within the last 30 days Yes, within the last 12 months Yes, more than 12 months ago No, never |
| DUIC_FREQ_01M | In the last 30 days, how often have you driven a motor vehicle (e.g. car, motorbike) within 2 hours of using cannabis? | 1 time  2–3 times  4–9 times  10–15 times more than 15 times, namely ___ I don't know |
| DUIC_MIXEDUSE | If you now think of the situation or situations in which you drove a motor vehicle (e.g. car, motorbike) within 2 hours of using cannabis – did you also use alcohol or other drugs (e.g. amphetamines, cocaine) in addition to cannabis?  In situations where I drove a motor vehicle within 2 hours of consuming cannabis, I had: | Only used cannabis  Occasionally used alcohol or other drugs in addition to cannabis Mostly used alcohol or other drugs in addition to cannabis Always used alcohol or other drugs in addition to cannabis I don't know |
| DRIVERLICENSE | Do you have a valid driving license for a car or motorbike in Germany/Austria? | Yes No, never had a license No, but used to have a license |
| EDUCATION_DE | What is your highest educational qualification? | Left school without a qualification Secondary school leaving certificate (9th grade) or polytechnic secondary school in the GDR (8th or 9th grade) Realschulabschluss (intermediate school leaving certificate, 10th grade) or GDR polytechnic secondary school (10th grade) Entrance qualification for universities of applied sciences, technical secondary school certificate General or subject-specific higher education entrance qualification/Abitur (Gymnasium or EOS, also EOS with apprenticeship) Still a student Another qualification, namely____ |
| GENDER | What is your gender? | Male Female Diverse |
| HOBBYS | In the last 12 months, how often have you done the following leisure activities?  Watching television, films, series (also on the Internet) Go for a walk Playing games of chance (e.g. slot machines, lotteries, poker, sports betting, etc.) Doing sport yourself Attend concerts or go to clubs/discos Being active in a club | Not at all Less than once a month At least once a month At least once a week (Almost) daily |
| MEDICALUSE_01 | In the last 12 months, have you used cannabis exclusively for recreational use or (also) for medical purposes (to treat an illness or alleviate symptoms)? | Exclusively for medical purposes For medical and recreational use Exclusively for recreational use |
| MEDICALUSE_02 | Have you been prescribed medical cannabis (flowers or preparations such as Sativex or dronabinol) by a doctor in the last 12 months? | Yes, I have been prescribed medical cannabis by a doctor (paid for by health insurance or as a self-payer) No, I do not have a doctor's prescription for medicinal cannabis |
| SOCIALDESIRE  Social Desirability – Gamma Short Scale (KSE-G; German Original)^15^  * items belong to the Minimisation of Negative Qualities subscale (NQ–), Reliability: ω = .78 | The following statements may apply to you to a greater or lesser extent. For each statement, please indicate how much the statement applies to you.  *It has happened before that I have taken advantage of someone. Even when I'm stressed myself, I always treat others kindly and courteously. *Sometimes I only help someone if I can expect something in return. I always remain objective and dispassionate in arguments. *I have sometimes thrown rubbish in the countryside or on the street. When I talk to someone, I always listen carefully. | Does not apply at all Applies a little Applies somewhat Applies quite a bit Applies completely |
| STIGMA_01 | When others find out about my cannabis use, they think badly of me. | Strongly disagree 1 - 2 - 3 - 4 - 5 Strongly agree |
| STIGMA_02 | I feel ashamed when other people find out about my cannabis use. | Strongly disagree 1 - 2 - 3 - 4 - 5 Strongly agree |
| SUBSTANCES | How often have you consumed the following products in the last 12 months?  Fresh fruit Fresh vegetables or salad Alcohol Tobacco E-cigarettes *Cannabis (weed, marijuana, hashish) Fast food Meat Fish Highly sugary drinks such as sweetened fruit juice drinks, lemonade, cola etc. Sweets, chocolate, cakes  *Footnote "Cannabis": THC-containing products, such as weed, hashish, liquid and solid concentrates, edible products, NOT synthetic cannabinoids or CBD cannabis with less than 0.3% THC-content | Not at all Less than once a month At least once a month At least once a week (Almost) daily |

Note. All items were originally in German and translated into English for presentation. The full questionnaire, including filter logic and additional items, is available online (<https://osf.io/72nyf/>).

# **Supplement S5: Social desirability bias in measuring DUIC**

Self-reported DUIC may be biased by social desirability. As our analysis focus on changes in DUIC over time, it is not the absolute level of underreporting that threatens validity, but rather changes in the strength of this bias post-legalisation, especially as public attitudes and stigmatisation toward people using cannabis may change. Therefore, we examined (1) whether DUIC was associated with social desirability at all, (2) whether this association changed following legalisation, and (3) whether stigma changed post-legalisation.

**S5.1 Social desirability**

To assess social desirability, we used the *Minimization of Negative Qualities* (NQ–) subscale of the Social Desirability – Gamma Short Scale (KSE-G)^15^, which has been validated for the adult population in Germany,

been shown to correlate with self-reported deviant behaviors such as driving above the legal alcohol limit.^15^ The NQ– subscale consists of three items (*“I have taken advantage of people in the past.”, “Sometimes I only help someone if I can expect something in return.”, “I have thrown rubbish into the countryside or onto the street“*) assessing the tendency to deny socially undesirable traits. Responses were given on a 5-point Likert scale (1=*does not apply at all* to 5 = *applies completely*). After reverse-coding these items, we computed the mean score across the three items, with higher values indicating stronger minimisation of negative qualities, i.e. higher social desirability. Z-standardised scores were calculated based on age (18–35 / 36–65), education (low / medium / high), and gender (female / male) using German population norms.^15^

We estimated a logistic regression model with past 12-month DUIC (0=no, 1=yes) as the outcome variable and included NQ– (z-scores), time (0=5 to 6 months before legalisation (t₀), 1=7 to 9 months after legalisation (t₁)), and their interaction as predictors using Sample 2 (data from Germany only, as the KSE-G was not included in the Austrian survey version).

The main effect of NQ– was significant (*OR*=0·60, 95% *CI* 0·48–0·74, *p*<·001), while the interaction between NQ– and time was not (Table S5.1). Thus, although some absolute underreporting may exist, the absence of a statistically significant time-varying effect indicates that our DUIC analysis is unlikely to be confounded by shifts in social desirability bias.

**Table S5.1 Logistic regression model predicting past 12-month DUIC from social desirability (NQ–) and survey wave (Sample 2, Germany only)**

|  | ***OR* (95% CI)** | ***p-*value** |
| --- | --- | --- |
| *NQ–* (z-scores) | 0·60 (0·48–0·74) | < ·001 |
| *Time*: t_1_ (reference: t_0_) | 0·96 (0·68–1·36) | ·818 |
| *NQ–* x *Time* | 1·12 (0·87–1·46) | ·380 |

Note. Odds Ratios (OR) and 95% Confidence Intervals (CI) are reported from unadjusted and unweighted binomial logistic regression model. NQ–=Minimization of Negative Qualities (subscale of the KSE-G). R^2^=·07 (Nagelkerke); χ^2^(3)=50·7, *p*<·001. Sample sizes (Sample 2, Germany only): *n*_t0_=392, *n*_t1_=588; t₀: Nov–Dec 2023, t₁: Nov 2024–Jan 2025.

**S5.2 Stigma**

Stigma was addressed in the present study (not included in the Austrian survey version) utilising two questions, one relating to perceived external stigmatisation (“*When others find out about my cannabis use, they think badly of me.*”) and one to self-stigma (“*I feel ashamed when other people find out about my cannabis use.*”). Responses were given on a 5-point Likert scale (1 = *Strongly disagree* to 5 = *Strongly agree*). We examined whether the perceived external and self-stigma changed from t_0_ to t_1_ using chi-square tests. Our analysis showed no significant changes between the survey waves (see Table S5.2), suggesting that potential changes in experiences of stigmatisation are unlikely to have biased DUIC responses across time points.

**Table S5.2 Stigmatisation by survey wave (Sample 2, Germany only)**

|  | **n (%)** | | **χ² (df)** | ***p* value** |
| --- | --- | --- | --- | --- |
|  | **t_0_** | **t_1_** |  |  |
| **Perceived stigmatisation** |  |  | 2·39 (2) | ·302 |
| yes | 106 (26·8) | 131 (24·0) |  |  |
| neither/nor | 134 (33·9) | 183 (31·9) |  |  |
| no | 153(39·4) | 273 (44·1) |  |  |
| **Self-stigma** |  |  | 0·13 (2) | ·936 |
| yes | 74 (19·6) | 104 (19·0) |  |  |
| neither/nor | 98 (24·7) | 124 (24·1) |  |  |
| no | 217 (55·7) | 358 (56·9) |  |  |

Note. Yes = *strongly agree/agree*, neither/nor = *neither agree nor disagree*, no = *disagree/strongly disagree*. Sample sizes (Sample 2, Germany only): *n*_t0_=393; *n*_t1_=587; t₀: Nov–Dec 2023, t₁: Nov 2024–Jan 2025.

# Supplement S6: Selection of covariates for DiD models

**Table S6.1. Bivariate associations between sociodemographic and behavioral covariates and cannabis use pre-legalisation (t₀, German and Austrian sample combined)**

|  | **Past 12-month cannabis use, *n* (% yes)** | **χ² (df)** | ***p-*value** |
| --- | --- | --- | --- |
| **Sociodemographic covariates** |  |  |  |
| **Gender** |  | 94·5 (1) | **<·001** |
| male | 625 (14·9) |  |  |
| female | 383 (8·3) |  |  |
| **Age group** |  | 318·1 (3) | **<·001** |
| 18–24 | 187 (19·8) |  |  |
| 25–34 | 372 (18·8) |  |  |
| 35–44 | 240 (12·4) |  |  |
| 45–64 | 209 (5·3) |  |  |
| **Degree of urbanisation** |  | 101·3 (2) | **<·001** |
| city | 553 (15·6) |  |  |
| town/suburb | 308 (9·2) |  |  |
| rural | 147 (7·8) |  |  |
| **Education** |  | 70·9 (2) | **<·001** |
| low | 65 (14·3) |  |  |
| medium | 476 (9·1) |  |  |
| high | 465 (15·0) |  |  |
| **Behavioral covariates** |  |  |  |
| **Frequency of alcohol use** |  | 256·5 (4) | **<·001** |
| never | 97 (4·8) |  |  |
| less than monthly | 183 (8·7) |  |  |
| monthly | 256 (12·5) |  |  |
| weekly | 337 (15·7) |  |  |
| (near) daily | 135 (26·5) |  |  |
| **Frequency of gambling** |  | 431·3 (4) | **<·001** |
| never | 346 (7·0) |  |  |
| less than monthly | 215 (12·3) |  |  |
| monthly | 162 (16·8) |  |  |
| weekly | 200 (20·9) |  |  |
| (near) daily | 85 (46·4) |  |  |
| **Frequency of physical activity** |  | 97·8 (4) | **<·001** |
| never | 87 (6·2) |  |  |
| less than monthly | 107 (8·6) |  |  |
| monthly | 137 (11·9) |  |  |
| weekly | 400 (11·8) |  |  |
| (near) daily | 277 (17·0) |  |  |

Note. Unweighted values; *n* and % refer to participants with past 12-month cannabis use. Chi-square tests were conducted using baseline data (t₀) from Sample 1 (i.e., general population); *n*_t0_=8802, German and Austrian data were pooled. Covariates with *p*<·10 were retained for inclusion in the sensitivity analysis of the cannabis use difference-in-differences model.

**Table S6.2. Bivariate associations between sociodemographic and behavioral covariates and DUIC pre-legalisation (t₀, German and Austrian sample combined)**

|  | **Past 12-month DUIC, *n* (% yes)** | **χ² (df)** | ***p-*value** |
| --- | --- | --- | --- |
| **Sociodemographic covariates** |  |  |  |
| **Gender** |  | 4·1 (1) | **·044** |
| male | 91 (28·7) |  |  |
| female | 32 (19·8) |  |  |
| **Age group** |  | 16·9 (3) | **<·001** |
| 18–24 | 20 (24·7) |  |  |
| 25–34 | 63 (35·0) |  |  |
| 35–44 | 28 (22·4) |  |  |
| 45–64 | 12 (12·9) |  |  |
| **Driver’s license** |  | 13·8 (1) | **<·001** |
| yes | 111 (29·8) |  |  |
| no | 12 (11·3) |  |  |
| **Degree of urbanisation** |  | 1·4 (2) | ·501 |
| city | 70 (27·5) |  |  |
| town/suburb | 39 (25·0) |  |  |
| rural | 14 (20·6) |  |  |
| **Education** |  | 24·1 (2) | **<·001** |
| low | 1 (3·1) |  |  |
| medium | 44 (19·5) |  |  |
| high | 78 (35·5) |  |  |
| **Behavioral covariates** |  |  |  |
| **Frequency of cannabis use** |  | 0·8 (2) | ·672 |
| monthly | 42 (24·1) |  |  |
| weekly | 44 (28·2) |  |  |
| (near) daily | 37 (24·8) |  |  |
| **Frequency of alcohol use** |  | 5·8 (4) | ·223 |
| never | 7 (13·5) |  |  |
| less than monthly | 16 (24·2) |  |  |
| monthly | 28 (25·0) |  |  |
| weekly | 47 (28·1) |  |  |
| (near) daily | 25 (30·5) |  |  |
| **Frequency of gambling** |  | 48·8 (4) | **<·001** |
| never | 9 (6·3) |  |  |
| less than monthly | 22 (24·7) |  |  |
| monthly | 26 (29·9) |  |  |
| weekly | 47 (39·8) |  |  |
| (near) daily | 19 (44·2) |  |  |
| **Frequency of physical activity** |  | 4·8 (4) | ·306 |
| never | 5 (13·1) |  |  |
| less than monthly | 12 (24·0) |  |  |
| monthly | 15 (22·1) |  |  |
| weekly | 48 (27·4) |  |  |
| (near) daily | 43 (29·1) |  |  |

Note. Unweighted values; *n* and % refer to participants with past 12-month driving under the influence of cannabis (DUIC). Chi-square tests were conducted using baseline data (t₀) from Sample 2 (i.e., at least monthly cannabis users without medical exemption); *n*_t0_=479, German and Austrian data were pooled. Sociodemographic covariates with *p*<·10 were retained for inclusion in the main DUIC difference-in-differences model and behavioral covariates with *p*<·10 for inclusion in sensitivity analysis. Alcohol use frequency was included as a covariate due to its theoretical relevance and clear linear association with DUIC (13–30%), despite not reaching statistical significance (*p*=·223).

# Supplement S7: Minimum detectable effect

We conducted a simulation-based, post-hoc sensitivity analysis to determine the minimum detectable effect (MDE) for both DiD-analyses (H1: cannabis use and H2: DUIC). The MDE was defined as the smallest intervention effect that would achieve ≥80% statistical power at α=·05, given the observed sample sizes, baseline prevalences (t₀) and time trends in Austria (control group). Under the parallel trends assumption, expected prevalences for Germany at t₁ were derived by adding Austria’s observed temporal change between t₀ and t₁ to Germany’s baseline prevalence (+0·2 percentage points (pp) for cannabis use; +3·5 pp for DUIC). For a grid of hypothetical intervention effects—defined as absolute increases above the expected prevalence (cannabis use: 0–10 pp in 0·2-pp increments; DUIC: 0–40 pp in 0·2-pp increments)—we simulated 500 datasets per intervention effect size. For each simulation, binary outcomes were generated using random binomial draws conditional on country-specific sample sizes and prevalences:

- Austria (control group):
  - Cannabis use: p_t0_=0·094 (n _t0_=2,132); p _t1_=0·096 (n _t1_=2,102)
  - DUIC: p_t0_=0·128 (n _t0_=86); p _t1_=0·163 (n _t1_=92)
- Germany (intervention group):
  - Cannabis use: p_t0_=0·121 (n _t0_=6,670); p _t1_=p_t0_ + temporal trend + intervention effect (n _t1_=9,692)
  - DUIC: p_t0_=0·285 (n _t0_=393); p _t1_=p_t0_ + temporal trend + intervention effect (n _t1_=589)

For each simulated dataset, we estimated a logistic DiD regression model including the time × country interaction term (DiD-effect). Power was defined as the proportion of simulations in which the DiD-effect reached statistical significance (p<·05). The MDE was the smallest DiD-effect achieving ≥80% power.

**Table S7. Input data and minimum detectable effects at 80% statistical power for primary and secondary analysis**

|  | **DiD analysis: cannabis use (H1)** | **DiD analysis: DUIC (H2)** |
| --- | --- | --- |
| Observed t_0_ 12-months prevalence (Germany, %) | 12·1 | 28·5 |
| Observed t_0_ 12-months prevalence (Austria, %) | 9·4 | 12·8 |
| Expected t_1_ prevalence without intervention effect (Germany, %) | 12·3 | 32·0 |
| MDE (OR) | 1·40 | 3·68 |
| MDE (absolute, pp) | 4·2 | 33·6 |
| Achieved power at MDE (%) | 82·4 | 80·6 |

Note. MDE = minimum detectable effect; pp = percentage points; OR = odds ratio; DiD=difference-in-differences : H1/2 = hypothesis 1/2. Baseline prevalences are observed values (weighted for cannabis use and unweighted for DUIC). The expected German t_1_ prevalence under the parallel trends assumption (i.e., without intervention) was calculated by applying Austria's observed temporal trend to Germany's baseline. MDE (pp) denotes the absolute increase in Germany’s prevalence above the expected prevalence without intervention effect. Achieved power indicates the statistical power at the identified MDE. Sample sizes (Sample 1, cannabis use): Germany: *n*_t0_= 6,670, *n*_t1_= 9,692; Austria: *n*_t0_= 2,132; Sample sizes (Sample 2, DUIC, at least monthly cannabis users without medical exemption): Germany: *n*_t0_=393, *n*_t1_= 589, Austria: *n*_t0_=86, *n*_t1_=92.

# Supplement S8: Sample weighting

Post-stratification sample weights for Sample 1 were calculated as the inverse of the probability of participating in the survey within each stratum. The strata were defined by age (18–29, 30–39, 40–49, 50–64 years), gender (female, male), highest level of education (ISCED level 1/2, ISCED level ≥ 3), and place of residence (federal state). Stratum-specific population distributions were sourced from the Federal Statistical Offices of Germany and Austria. An iterative, multivariate weighting process was used, with multiple iterations aiming to progressively converge towards the desired proportions by simultaneously adjusting multiple attributes.

Sample weights ranged from 0·62–2·01 (AT, t_0_), 0·53–3·41(AT, t_1_), 0·42–1·96 (DE, t_0_), 0·47–3·30 (DE, t_1_).

# Supplement S9: Sensitivity analyses for DiD models

**Rationale for sensitivity analysis using a negative control outcomes**

As a robustness check, we conducted a sensitivity analysis using fish consumption and physical activity in the past 12 months as negative control outcomes, under the assumption that they are unaffected by cannabis policy but may share similar confounding structures with the main outcomes (fish consumption and cannabis use: e.g., age, gender, socioeconomic factors; physical activity and DUIC: e.g., age, gender, sensation seeking). Negative controls are suited to detect potential residual confounding (variations in sample characteristics between time points) or spurious associations in the DiD models.

Suitability criteria for our negative controls included (1) a theoretically similar confounding structure to the primary outcomes, (2) no expected effect of cannabis legalisation, (3) sufficient variability in the population, and (4) reference to the same 12-month recall period.

We examined fish consumption (0=never; 1=less than monthly/monthly/weekly/(near) daily; Item 'SUBSTANCES') in sample 1 (total population) and physical activity (0=never; 1=less than monthly/monthly/weekly/(near) daily; Item 'HOBBIES') in the past 12 months in sample 2 (at least monthly cannabis users). Two logistic DiD models were estimated, mirroring the specifications of the primary models described in Section 2.3.1: one in Sample 1 (weighted, analogous to the cannabis use model for H1) with past 12-month fish consumption as outcome variable, and one in Sample 2 (unweighted, but adjusted, analogous to the DUIC model for H2) with past 12-month physical activity as outcome variable, including the same covariates used in the DUIC analysis. A statistically significant interaction term (t₁ × Germany) would indicate potential confounding.

**Table S9.1. Results from sensitivity analyses: logistic regression models (difference-in differences) for past 12-month cannabis use and negative control outcome (Hypothesis 1, Sample 1)**

|  | **(*a*)*OR* (95% CI)** | ***p*-value** |
| --- | --- | --- |
| **Sensitivity analysis 1 (unweighted and unadjusted): Past 12-month cannabis use** | |  |
| *Time*: t_1_ (reference: t_0_) | 1·00 (0·81–1·24) | ·972 |
| *Country*: Germany (reference: Austria) | 1·45 (1·23–1·72) | **<·001** |
| t_1_ x Germany (DiD-effect) | 0·91 (0·72–1·15) | ·427 |
| **Sensitivity analysis 2 (unweighted, including sociodemographic covariates): Past 12-month cannabis use** |  |  |
| *Time*: t_1_ (reference: t_0_) | 0·99 (0·79–1·23) | ·923 |
| *Country*: Germany (reference: Austria) | 1·22 (1·03–1·45) | **·025** |
| t_1_ x Germany (DiD-effect) | 1·21 (0·95–1·54) | ·128 |
| *Gender*: female (reference: male) | 0·45 (0·41–0·50) | **<·001** |
| *Age group* (reference: 18–24 years): |  |  |
| 25–34 years | 0·90 (0·78–1·04) | ·148 |
| 35–44 years | 0·52 (0·44–0·60) | **<·001** |
| 45–64 years | 0·19 (0·17–0·23) | **<·001** |
| *Degree of urbanisation* (reference: city): |  |  |
| town/suburb | 0·68 (0·61–0·75) | **<·001** |
| rural | 0·58 (0·51–0·67) | **<·001** |
| *Education* (reference: low): |  |  |
| medium | 0·68 (0·57–0·82) | **<·001** |
| high | 0·92 (0·76–1·11) | ·380 |
| **Sensitivity analysis 3 (unweighted, including sociodemographic and behavioral covariates): Past 12-month cannabis use** |  |  |
| *Time*: t_1_ (reference: t_0_) | 0·99 (0·79–1·24) | ·939 |
| *Country*: Germany (reference: Austria) | 1·27 (1·06–1·52) | **·009** |
| t_1_ x Germany (DiD-effect) | 1·23 (0·96–1·57) | ·103 |
| *Gender*: female (reference: male) | 0·65 (0·59–0·72) | **<·001** |
| *Age group* (reference: 18–24 years): |  |  |
| 25–34 years | 0·82 (0·70–0·95) | **·008** |
| 35–44 years | 0·44 (0·38–0·52) | **<·001** |
| 45–64 years | 0·17 (0·14–0·20) | **<·001** |
| *Degree of urbanisation* (reference: city): |  |  |
| town/suburb | 0·71 (0·64–0·79) | **<·001** |
| rural | 0·59 (0·52–0·68) | **<·001** |
| *Education* (reference: low): |  |  |
| medium | 0·55 (0·46–0·67) | **< 001** |
| high | 0·66 (0·54–0·80) | **<·001** |
| *Frequency of alcohol use* (reference: never): |  |  |
| less than monthly | 1·39 (1·19–1·64) | **<·001** |
| monthly | 1·99 (1·70–2·32) | **<·001** |
| weekly | 2·52 (2·16–2·94) | **<·001** |
| (near) daily | 4·40 (3·57–5·41) | **<·001** |
| *Frequency of gambling* (reference: never): |  |  |
| less than monthly | 1·45 (1·28–1·65) | **<·001** |
| monthly | 2·22 (1·93–2·55) | **<·001** |
| weekly | 2·52 (2·19–2·90) | **<·001** |
| (near) daily | 4·86 (3·75–6·27) | **<·001** |
| *Frequency of physical activity* (reference: never): |  |  |
| less than monthly | 1·22 (1·00–1·47) | **·045** |
| monthly | 1·43 (1·19–1·73) | **<·001** |
| weekly | 1·17 (0·99–1·38) | ·053 |
| (near) daily | 1·36 (1·14–1·63) | **<·001** |
| **Negative control analysis (weighted): Past 12-month fish consumption** |  |  |
| *Time*: t_1_ (reference: t_0_) | 0·80 (0·66–0·97) | **·021** |
| *Country*: Germany (reference: Austria) | 0·84 (0·71–0·98) | **·026** |
| t_1_ x Germany (DiD-effect) | 1·21 (0·98–1·50) | ·075 |
| **Germany-only analysis (weighted): Past 12-month cannabis use** |  |  |
| *Time*: t_1_ (reference: t_0_) | 1·22 (1·11–1·34) | **<·001** |

Note. DiD=difference-in-differences. Sample sizes (Sample 1): Germany: *n*_t0_= 6,670, *n*_t1_= 9,692; Austria: *n*_t0_= 2,132, *n*_t1_= 2,102. t_0_: Nov–Dec 2023, t_1_: Nov 2024–Jan 2025. Sensitivity analysis 1: R^2^=·003 (Nagelkerke); χ^2^(3)=33·4, *p*<·001. Sensitivity analysis 2: R^2^=·126 (Nagelkerke); χ^2^(11)=1341·7, *p*<·001. Sensitivity analysis 3: R^2^=·192 (Nagelkerke); χ^2^(23)=2083·3, *p*<·001. Negative control analysis: R^2^=·001 (Nagelkerke); χ^2^(3) =8·0, *p*=·045. Germany-only analysis: R^2^=·002 (Nagelkerke); χ^2^(1)=18·0, *p*<·001. All models had generalized variance inflation factors below 1·22, except for *Country, Time* and the interaction *Country*x*Time*, which is expected as interactions are inherently correlated with the main effects. Adjusted odds ratios (aORs) are presented for sensitivity analyses 2 and 3; unadjusted ORs are presented for all other analyses.

**Table S9.2. Results from sensitivity analyses: logistic regression models (difference-in differences) for past 12-month DUIC, past 30-days DUIC and negative control outcome (Hypothesis 2, Sample 2)**

|  | **(*a*)*OR* (95% CI)** | ***p*-value** |
| --- | --- | --- |
| **Sensitivity Analysis 1 (unadjusted): Past 12-month DUIC** |  |  |
| *Time*: t_1_ (reference: t_0_) | 1·33 (0·58–3·15) | ·508 |
| *Country*: Germany (reference: Austria) | 2·72 (1·45–5·58) | **·003** |
| t_1_ x Germany (DiD-effect) | 0·69 (0·28–1·67) | ·417 |
| **Sensitivity Analysis 2 (adjusted for sociodemographic and behavioral covariates): Past 12-month DUIC** |  |  |
| *Time*: t_1_ (reference: t_0_) | 1·61 (0·68–3·94) | ·282 |
| *Country*: Germany (reference: Austria) | 2·10 (1·08–4·43) | **·038** |
| t_1_ x Germany (DiD-effect) | 0·73 (0·29–1·83) | ·507 |
| *Gender*: female (reference: male) | 0·88 (0·64–1·20) | ·422 |
| *Driver’s license*: yes (reference: no) | 1·84 (1·24–2·77) | **·003** |
| *Age group* (reference: 18–24 years): |  |  |
| 25–34 years | 1·12 (0·72–1·75) | ·613 |
| 35–44 years | 0·75 (0·47–1·20) | ·223 |
| 45–64 years | 0·48 (0·28–0·80) | **·005** |
| *Education* (reference: low): |  |  |
| medium | 1·95 (0·96–4·37) | ·081 |
| high | 2·54 (1·23–5·78) | **·017** |
| *Frequency of alcohol use* (reference: never): |  |  |
| less than monthly | 1·03 (0·58–1·85) | ·912 |
| monthly | 0·82 (0·48–1·41) | ·468 |
| weekly | 0·95 (0·57–1·59) | ·836 |
| (near) daily | 1·23 (0·69–2·21) | ·482 |
| *Frequency of gambling* (reference: never): |  |  |
| less than monthly | 2·14 (1·34–3·42) | **·002** |
| monthly | 2·94 (1·90–4·60) | **<·001** |
| weekly | 3·35 (2·17–5·24) | **<·001** |
| (near) daily | 4·38 (2·43–7·93) | **<·001** |
| **Sensitivity Analysis 3 (adjusted for sociodemographic covariates): Past 30-day DUIC** |  |  |
| *Time*: t_1_ (reference: t_0_) | 1·27 (0·43–3·83) | ·667 |
| *Country*: Germany (reference: Austria) | 1·31 (0·59–3·33) | ·532 |
| t_1_ x Germany (DiD-effect) | 0·97 (0·30–3·11) | ·965 |
| *Gender*: female (reference: male) | 0·66 (0·43–1·00) | ·052 |
| *Driver’s license*: yes (reference: no) | 3·19 (1·76–6·29) | **<·001** |
| *Age group* (reference: 18–24 years): |  |  |
| 25–34 years | 0·82 (0·48–1·45) | ·482 |
| 35–44 years | 0·54 (0·30–1·00) | **·048** |
| 45–64 years | 0·39 (0·20–0·75) | **·005** |
| *Education* (reference: low): |  |  |
| medium | 2·93 (1·03–12·32) | ·079 |
| high | 2·28 (0·78–9·74) | ·185 |
| **Negative control analysis (adjusted for sociodemographic covariates): Past 12-month physical activity** |  |  |
| *Time*: t_1_ (reference: t_0_) | 1·04 (0·36–2·90) | ·937 |
| *Country*: Germany (reference: Austria) | 1·25 (0·50–2·84) | ·605 |
| t_1_ x Germany (DiD-effect) | 0·63 (0·20–1·98) | ·422 |
| *Gender*: female (reference: male) | 0·84 (0·56–1·26) | ·399 |
| *Driver’s license*: yes (reference: no) | 1·37 (0·87–2·14) | ·166 |
| *Age group* (reference: 18–24 years): |  |  |
| 25–34 years | 0·55 (0·21–1·30) | ·197 |
| 35–44 years | 0·24 (0·09–0·53) | **·001** |
| 45–64 years | 0·11 (0·04–0·25) | **<·001** |
| *Education* (reference: low): |  |  |
| medium | 2·50 (1·40–4·40) | **·001** |
| high | 6·72 (3·37–13·39) | **<·001** |

Note. DiD=difference-in-differences. DUIC=driving under the influence of cannabis, t₀: Nov–Dec 2023, t₁: Nov 2024–Jan 2025. Sample sizes (Sample 2, at least monthly cannabis users without medical exemption): Germany: *n*_t0_=393, *n*_t1_= 589, Austria: *n*_t0_=86, *n*_t1_=92. Sensitivity analysis 1: R^2^=·019 (Nagelkerke); χ^2^(3)=15·3, *p*<·01. Sensitivity analysis 2: R^2^=·171 (Nagelkerke); χ^2^(18)=143·0, *p*<·001. Sensitivity analysis 3: R^2^=·065 (Nagelkerke); χ^2^(10)=37·5, *p*<·001. Negative control analysis: R^2^=·172 (Nagelkerke); χ^2^(10) =102·7, *p*<·001. All models had generalized variance inflation factors below 1·34, except for *Country, Time* and the interaction *Country*x*Time*, which is expected as interactions are inherently correlated with the main effects. Unadjusted odds ratios (OR) are presented for sensitivity analysis 1; adjusted odds ratios (aOR) are presented for all other analyses.

**Table S9.3. Results from sensitivity analyses: logistic regression model (difference-in-differences) for past 12-month DUIC in Sample 1**

|  | ***aOR* (95% CI)** | ***p*-value** |
| --- | --- | --- |
| **Past 12-month DUIC (adjusted for sociodemographic covariates)** |  |  |
| *Time*: t_1_ (reference: t_0_) | 1·44 (0·74–2·86) | ·290 |
| *Country*: Germany (reference: Austria) | 2·38 (1·43–4·27) | **·002** |
| t_1_ x Germany (DiD-effect) | 0·95 (0·46–1·92) | ·886 |
| *Gender*: female (reference: male) | 0·31 (0·24–0·38) | **<·001** |
| *Age group* (reference: 18–24 years): |  |  |
| 25–34 years | 1·14 (0·82–1·60) | ·442 |
| 35–44 years | 0·61 (0·43–0·88) | **·007** |
| 45–64 years | 0·14 (0·09–0·21) | **<·001** |
| *Degree of urbanisation* (reference: city): |  |  |
| town/suburb | 0·80 (0·63–1·01) | ·066 |
| rural | 0·80 (0·58–1·07) | ·145 |
| *Education* (reference: low): |  |  |
| medium | 1·27 (0·73–2·43) | ·438 |
| high | 2·50 (1·44–4·79) | **·003** |

Note. DiD = difference-in-differences. Sample sizes (Sample 1): Germany: *n*_t0_=6,670; *n*_t1_=9,692; Austria *n*_t0_=2,132; *n*_t1_=2,102. t_0_: Nov–Dec 2023, t_1_: Nov 2024–Jan 2025. Missing DUIC values for less than monthly cannabis users in Germany t₀ (not surveyed) were imputed using a logistic regression model estimated from corresponding t₁ data. The imputation model included age group, gender, education, degree of urbanisation, frequency of gambling, alcohol, and physical activity as predictors. Predicted probabilities were converted into binary outcomes via stochastic draws from a Bernoulli distribution. This approach relies on the assumption that DUIC prevalence in this subgroup remained stable and smooths potential changes due to legalisation. Possession of a driver’s license was not surveyed in the full sample and therefore not added as a covariate. For medical-only users with a prescription, DUIC was set to 0. R^2^=·13 (Nagelkerke); χ^2^(11)=440·2, *p*<·001. All generalized variance inflation factors were below 1·07, except for *Country, Time* and the interaction *Country*x*Time*, which is expected as interactions are inherently correlated with the main effects. Weighted past 12-month DUIC prevalences based on the aforementioned assumptions: Germany t_0_: 2·0% (95% *CI* 1·7–2·4), t_1_: 2·8% (95% *CI* 2·4–3·2); Austria t_0_: 0·8% (95% *CI* 0·4–1·3), t_1_: 1·1% (95% *CI* 0·6–1·6)

# Supplement S10: DUIC among at least monthly medical-only cannabis users with a prescription

Among at least monthly cannabis users (Germany: *n*_t0_=498, *n*_t1_=687; Austria: *n*_t0_=100, *n*_t1_=99), there were *n*_t0_=105 and *n*_t1_=98 respondents in Germany, and *n*_t0_=14 and *n*_t1_=7 in Austria who reported using cannabis exclusively for medical purposes (Item ‘MEDICALUSE.01’; see Table S4) and held a prescription (Item ‘MEDICALUSE.02’; see Table S4). As the Austrian subgroup was too small for reliable estimates of DUIC, prevalence is reported for Germany only. Prior to legalisation (t_0_), the 12-month prevalence of DUIC among medical-only cannabis users with a prescription was 61·9% (95% *CI* 52·4–71·4), decreasing to 43·9% (95% *CI* 34·7–54·1) following legalisation (t_1_).

# Supplement S11: Sensitivity analyses for exploratory analysis: DUIC episodes by cannabis use frequency and involvement of alcohol or other drugs

**Figure S11.1. Sensitivity analysis: Distribution of cannabis users, DUIC(–) and DUIC(+) episodes by cannabis use frequency (German data only).**

DUIC(–) = driving under the influence of cannabis only, DUIC(+) = driving under the influence of cannabis in combination with alcohol or other drugs. *n* past year cannabis users = 988 (excluding medical-only users with a prescription), *n* DUIC(–) episodes = 362, *n* DUIC(+) episodes = 95, from *n* = 74 respondents (Sample 3 restricted to German data only, post-legalisation).


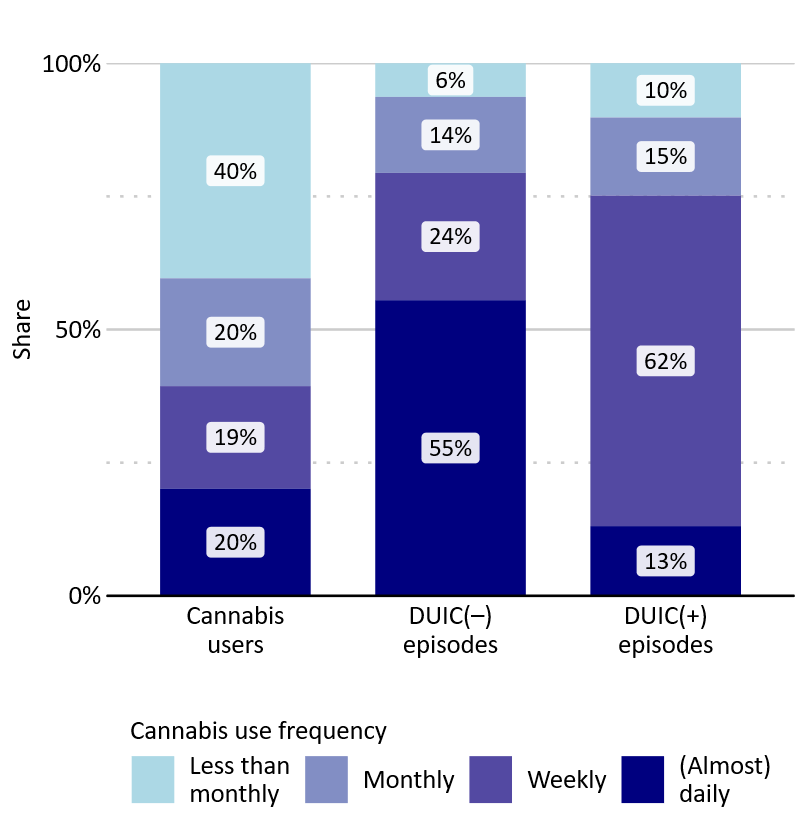


**Figure S11.2. Sensitivity analysis: Distribution of cannabis users, DUIC(–) and DUIC(+) episodes by cannabis use frequency, using alternative quantifications of ‘*occasionally used alcohol or other drugs in addition to cannabis*’ (60% of episodes classified as DUIC(–)) and ‘*mostly used alcohol or other drugs in addition to cannabis*’ (40% of episodes classified as DUIC(–)).**

DUIC(–) = driving under the influence of cannabis only, DUIC(+) = driving under the influence of cannabis in combination with alcohol or other drugs. *n* past year cannabis users = 1160 (excluding pure medical users with a prescription), *n* DUIC(–) episodes = 388, *n* DUIC(+) episodes = 112, from *n* = 84 respondents (Sample 3, post-legalisation, German and Austrian data are pooled).
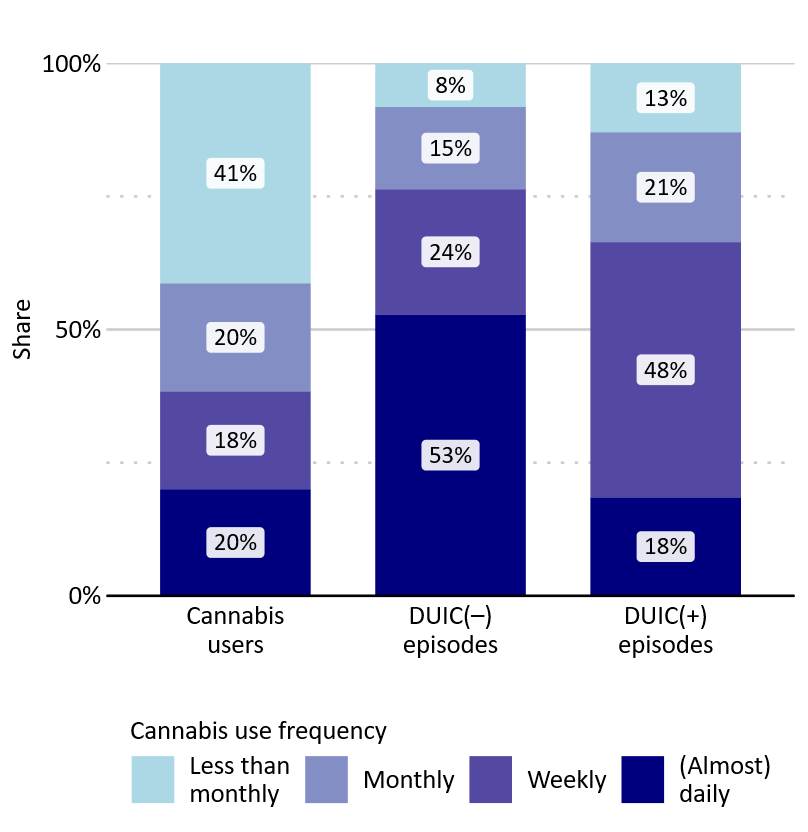


**Figure S11.3. Sensitivity analysis: Distribution of cannabis users, DUIC(–) and DUIC(+) episodes by cannabis use frequency, using alternative quantifications of ‘*occasionally used alcohol or other drugs in addition to cannabis*’ (90% of episodes classified as DUIC(–)) and ‘*mostly used alcohol or other drugs in addition to cannabis*’ (10% of episodes classified as DUIC(–)).**

DUIC(–) = driving under the influence of cannabis only, DUIC(+) = driving under the influence of cannabis in combination with alcohol or other drugs. *n* past year cannabis users=1160 (excluding pure medical users with a prescription), *n* DUIC(–) episodes=397, *n* DUIC(+) episodes=103, from *n*=84 respondents (Sample 3, post-legalisation, German and Austrian data are pooled).
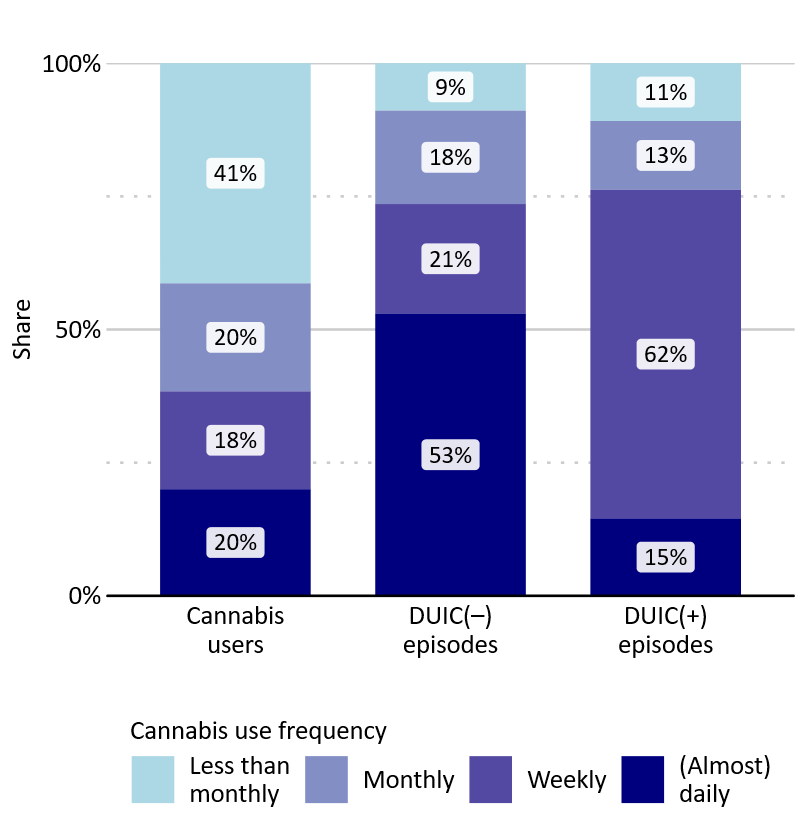


# References

1. ESPAD Group. Key findings from the 2024 European School Survey Project on Alcohol and Other Drugs (ESPAD) [Internet]. European Union Drugs Agency, Lisbon; 2025. Available from: https://www.euda.europa.eu/publications/data-factsheets/espad-2024-key-findings_en

2. Olderbak S, Möckl J, Manthey J, Lee S, Rehm J, Hoch E, et al. Trends and projection in the proportion of (heavy) cannabis use in Germany from 1995 to 2021. Addiction. 2024 Feb;119(2):311–21.

3. Olderbak S, Hollweck R, Krowartz EM, Möckl J, Hoch E. Psychoactive Substance Use in Germany: Findings From the Epidemiological Survey of Substance Abuse (ESA) in 2024. Dtsch Arztebl Int. 2025 Nov 14;(Forthcoming):arztebl.m2025.0157.

4. European Union Drugs Agency. Table EDR25-GPS-2: Prevalence of drug use in Europe, trends [Internet]. 2025. Available from: https://www.euda.europa.eu/publications/european-drug-report/2025/cannabis_en

5. Federal Statistical Office (Destatis). Population: Germany, reference date (Code: 12411-0001) [Internet]. 2025. Available from: https://www-genesis.destatis.de/genesis/online?code=12411-0001

6. Statistics Austria (Statistik Austria). Demographic Yearbook 2023 [Internet]. Vienna, Austria: Verlag Österreich GmbH for Statistics Austria; 2024. Available from: https://www.statistik.at/fileadmin/user_upload/Demo-JB-2023_Web-barrierefrei.pdf

7. Statistics Austria (Statistik Austria). Population at beginning of year / quarter [Internet]. Available from: https://www.statistik.at/statistiken/bevoelkerung-und-soziales/bevoelkerung/bevoelkerungsstand/bevoelkerung-zu-jahres-/-quartalsanfang

8. Federal Statistical Office (Destatis). Accidents involving personal injury, Germany (Table 46241‑0010) [Internet]. Available from: https://www-genesis.destatis.de/datenbank/online/table/46241-0010/search/s/NDYyNDEtMDAxMA==

9. Statistics Austria. Straßenverkehrsunfälle mit Personenschaden. Jahresergebnisse 2024 [Road traffic accidents involving personal injury. Annual results 2024] [Internet]. Vienna: Statistik Austria; 2025 Apr. Report No.: Statistik im Fokus 4.3. Available from: https://www.statistik.at/fileadmin/publications/SB_4-3_Unfaelle-Strasse_2024.pdf

10. RKI. Regional health differences – developing a socioeconomic deprivation index for Germany. 2017 [cited 2025 July 23]; Available from: http://edoc.rki.de/docviews/abstract.php?lang=ger&id=5130

11. Rauschert C, Möckl J, Wilms N, Hoch E, Kraus L, Olderbak S. Kurzbericht Epidemiologischer Suchtsurvey 2021. Tabellenband: (problematischer) Konsum illegaler Drogen und multiple Drogenerfahrung nach Geschlecht und Alter im Jahr 2021 [Internet]. IFT Institut für Therapieforschung; 2023 Feb. Available from: https://www.esa-survey.de/fileadmin/user_upload/Literatur/Berichte/ESA_2021_Tabellen_illegale_Drogen.pdf

12. Buth S, Meyer G, Rosenkranz M, Kalke J. Glücksspielteilnahme und glücksspielbezogene Probleme in der Bevölkerung. 2023;

13. Kotz D, Klosterhalfen S. Prävalenz Tabakrauchen [Internet]. Deutsche Rauchbefragung. 2024 [cited 2025 Apr 18]. Available from: https://www.debra-study.info/

14. Strizek J, Martin B, Puhm A, Schwarz T, Uhl A. Repräsentativerhebung zu Konsum‐ und Verhaltensweisen mit Suchtpotenzial. Wien: Gesundheit Österreich; 2021.

15. Kemper CJ, Beierlein C, Bensch D, Kovaleva A, Rammstedt B. Soziale Erwünschtheit-Gamma (KSE-G). Zusammenstellung sozialwissenschaftlicher Items und Skalen (ZIS) [Internet]. 2014 [cited 2024 Jan 9]; Available from: https://zis.gesis.org/DoiId/zis186
