# Supplementary material for: Short-term effects of cannabis legalisation in Germany on driving under the influence of cannabis: a difference-in-differences analysis using Austria as a control
Source: Lancet Reg Health Eur. 2026 Jan 23;63:101593. doi: 10.1016/j.lanepe.2026.101593 (PMC12860694; doi:10.1016/j.lanepe.2026.101593)
Supplement: German Translation [file mmc2.docx]

**Editor disclaimer:** This translation in German was submitted by the authors and we reproduce it as supplied. It has not been peer reviewed. Our editorial processes have only been applied to the original abstract in English, which should serve as reference for this manuscript.

**Zusammenfassung**

**Hintergrund:** Deutschland hat im April 2024 den Besitz und Anbau von Cannabis für Erwachsene legalisiert und im August 2024 neue gesetzliche THC-Grenzwerte für den Straßenverkehr festgelegt. Diese Studie untersucht die kurzfristigen Auswirkungen auf (1) den Cannabiskonsum und (2) das Fahren unter Cannabiseinfluss (*driving under the influence of cannabis*, DUIC) sowie (3) den Anteil an DUIC-Fahrten, bei denen zusätzlich Alkohol oder andere Drogen konsumiert wurden (DUIC(+)).

**Methoden:** Es wurden wiederholt-querschnittliche Befragungen in Deutschland und Österreich (Kontrollgruppe) vor (t₀: Nov.–Dez. 2023) und nach der Legalisierung (t₁: Nov. 2024–Jan. 2025) durchgeführt. Mittels *Difference-in-Differences*-Ansatz (DiD) wurde geprüft, ob sich die 12-Monats-Prävalenz 1) des Cannabiskonsums unter Erwachsenen im Alter von 18 bis 64 Jahren (Deutschland: n_t₀_=6.670, n_t₁_=9.692; Österreich: n_t₀_=2.132, n_t₁_=2.102) und 2) des DUIC unter mindestens monatlich Cannabiskonsumierenden (ausgenommen medizinischer Konsum; Deutschland: n_t₀_=393, n_t₁_=589; Österreich: n_t₀_=86, n_t₁_=92) im Zuge der Legalisierung verändert. Außerdem wurde zu t₁ analysiert, in wie vielen DUIC-Fahrten nur Cannabis und wie viele zusätzlich Alkohol oder andere Drogen konsumiert wurden (DUIC(+)), getrennt nach Konsumhäufigkeit.

**Ergebnisse**: In Deutschland stieg der Cannabiskonsum von 12,1 % auf 14,4 %, wobei sich dieser Anstieg statistisch nicht signifikant von Österreich unterschied (DiD-Effekt: OR=1,18, 95 % CI 0,95–1,48, p=·141, gewichtet). Unter den mindestens monatlich Konsumierenden nahm DUIC leicht von 28,5 % auf 26,8 % ab (ungewichtet), ebenfalls ohne signifikanten Unterschied zu Österreich (DiD-Effekt: aOR = 0,68, 95 % KI 0,27–1,68, p = 0,408. Zu t₁ machten DUIC(+)-Fahrten 21,5 % aller DUIC-Fahrten aus. DUIC(–) war bei täglich Konsumierenden am häufigsten, DUIC(+) bei wöchentlich Konsumierenden.

**Interpretation**: Acht Monate nach der Legalisierung wurden keine signifikanten kurzfristigen Auswirkungen auf den Cannabiskonsum oder DUIC beobachtet. Das mit einem höheren Verkehrsrisiko verbundene DUIC(+) wurde am häufigsten von wöchentlichen Konsumierenden berichtet. Für eine umfassende Evaluation der Legalisierung ist eine weitere Beobachtung von DUIC und Verkehrsdaten notwendig.
